# Scaling Up DNA Origami Lattice Assembly

**DOI:** 10.1002/chem.202100784

**Published:** 2021-05-04

**Authors:** Yang Xin, Boxuan Shen, Mauri A. Kostiainen, Guido Grundmeier, Mario Castro, Veikko Linko, Adrian Keller

**Affiliations:** ^1^ Technical and Macromolecular Chemistry Paderborn University Warburger Str. 100 33098 Paderborn Germany; ^2^ Biohybrid Materials Department of Bioproducts and Biosystems Aalto University P. O. Box 16100 00076 Aalto Finland; ^3^ Grupo Interdisciplinar de Sistemas Complejos and Instituto de Investigación Tecnológica Universidad Pontificia Comillas de Madrid Madrid 28015 Spain

**Keywords:** DNA origami, lattice formation, molecular lithography, self-assembly, topological analysis

## Abstract

The surface‐assisted hierarchical assembly of DNA origami nanostructures is a promising route to fabricate regular nanoscale lattices. In this work, the scalability of this approach is explored and the formation of a homogeneous polycrystalline DNA origami lattice at the mica‐electrolyte interface over a total surface area of 18.75 cm^2^ is demonstrated. The topological analysis of more than 50 individual AFM images recorded at random locations over the sample surface showed only minuscule and random variations in the quality and order of the assembled lattice. The analysis of more than 450 fluorescence microscopy images of a quantum dot‐decorated DNA origami lattice further revealed a very homogeneous surface coverage over cm^2^ areas with only minor boundary effects at the substrate edges. At total DNA costs of € 0.12 per cm^2^, this large‐scale nanopatterning technique holds great promise for the fabrication of functional surfaces.

## Graphical abstract

**The surface‐assisted assembly** of DNA origami lattices is a promising technique for fabricating functional surfaces. Here, the formation of a homogeneous DNA origami lattice on mica over a total surface area of 18.75 cm^2^ is demonstrated. Atomic force and fluorescence microscopy mapping reveal the high uniformity and homogeneity of the lattice over almost the entire surface area. The total DNA costs amount to €0.12 per cm^2^.

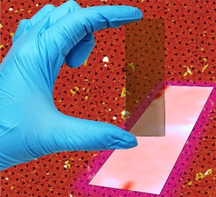

## Introduction

Already around the year 2000, several attempts were published that aimed at transforming single DNA molecules and larger DNA nanostructures into metallic nanowires.^[1]^ Since then, DNA‐based nanofabrication has come a long way.^[2]^ In particular the introduction of DNA origami,^[3]^ which enables the high‐yield synthesis of almost arbitrary nanoscale shapes has stimulated intense research efforts that, in the last few years, culminated in numerous strategies for transferring these DNA origami shapes into various organic[^4^, ^5^] and inorganic materials.[^6^, ^7^, ^8^] Although some of these attempts have resulted in new functional devices,[^8^, ^9^, ^10^, ^11^] their functionality so far relies on the properties of either a single nanostructure[^8^, ^9^, ^10^, ^11^] or a random ensemble of identical nanostructures.^[7]^ Up to now, however, it has been rather challenging to generate well‐defined arrangements of DNA origami nanostructures and transfer such templates into functional circuits or lattices with macroscopic dimensions to enable large‐scale device integration.

Large‐scale integration of nanostructures into macroscopic devices requires their controlled arrangement on solid surfaces to form designed circuits or regular lattices over macroscopic length scales. For DNA origami nanostructures, this may be achieved via directed adsorption onto prepatterned substrates^[12]^ or postadsorption manipulation.[^13^, ^14^] The former, however, typically requires rather sophisticated and time‐consuming preprocessing steps that may not be compatible with subsequent shape transfer. The latter, on the other hand, typically results only in rather limited control over placement and orientation of each individual DNA origami nanostructure in larger arrangements.

**Figure 1:**
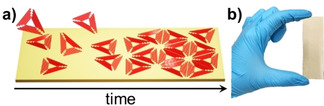
a) Schematic representation of the surface‐assisted hierarchical assembly of DNA origami lattices. b) Photograph of the 7.5×2.5 cm^2^ mica substrate.

As a rather straightforward method for the controlled arrangement of DNA nanostructures into highly regular 2D lattices, surface‐assisted hierarchical self‐assembly is receiving increasing attention.[^5^, ^15^, ^16^, ^17^, ^18^, ^19^, ^20^, ^21^, ^22^, ^23^, ^24^] Here, DNA nanostructures are adsorbed on mica surfaces or lipid bilayers under conditions that result in sufficient surface diffusion to enable their dynamic rearrangement (see Figure 1a). In this way, they can accommodate incoming DNA nanostructures from solution and optimize their packing on the surface, finally forming a close‐packed monolayer whose symmetry is completely dictated by the shape of the employed DNA nanostructures, while the crystal lattice of the underlying mica surface does not seem to play an important role.^[22]^ Such monolayers, which have already been used to direct the adsorption of different functional species into regular lattices,[^5^, ^16^, ^17^, ^21^] typically consist of highly‐ordered crystallites up to a few microns in size. However, these individual crystallites are also close‐packed and make up a polycrystalline lattice that can easily reach tens and hundreds of square microns.^[20]^ While DNA lattices of an astonishingly high order and regularity can be obtained under optimized assembly conditions,[^20^, ^23^] the lattice quality is highly sensitive toward experimental conditions and sample handling procedures. In particular, sample washing and drying may result in serious lattice damage, for instance from the crystallization of residual salt at the surface or removal of stabilizing cations from the DNA‐mica interface.^[16]^ Furthermore, previous studies have typically employed rather small mica substrates with dimensions ranging from 2 to about 15 mm[^16^, ^18^, ^24^] and analyzed only atomic force microscopy (AFM) snapshots few μm in size.[^5^, ^16^, ^18^, ^20^, ^22^, ^23^, ^24^] Therefore, it is unclear how well this strategy can be scaled up to large substrates in the centimeter range and how homogeneous the resulting lattices will be over these macroscopic length scales. Nevertheless, homogeneous surface coverage is an important prerequisite for numerous technological applications and has thus been a constant driver of innovation in other surface coating techniques such as layer‐by‐layer assembly for many years.^[25]^

In this work, we thus explore the scalability of surface‐assisted hierarchical DNA origami assembly and demonstrate the formation of a homogeneous DNA origami lattice on mica over a total surface area of 18.75 cm^2^ (see Figure 1b). Lattice order is assessed using AFM and quantified based on an automated topological analysis^[23]^ of AFM images recorded at different positions, demonstrating that essentially the same degree of order is obtained over the entire surface area. Furthermore, using quantum dot (QD) decoration of the assembled DNA origami lattice, lattice homogeneity over the entire surface area is mapped by fluorescence microscopy, which reveals the high uniformity of DNA origami packing and coverage over centimeter length scales. At total DNA costs of € 0.12 per cm^2^, DNA origami lattice assembly thus may enable the homogeneous nanopatterning of macroscopic surfaces with real‐world applications.

## Results and Discussion

In order to assemble DNA origami lattices over macroscopic length scales, we employed an established protocol that was optimized in one of our previous studies by monitoring the dynamics of DNA origami lattice assembly *in situ* by high‐speed AFM.^[20]^ It relies on the exchange of Mg^2+^ salt bridges at the mica‐DNA interface by Na^+^ ions, which results in enhanced surface mobility of the DNA origami triangles and the formation of a hexagonally ordered lattice. While maintaining the originally reported ion concentrations, *i. e*., 10 mM Mg^2+^ and 75 mM Na^+^, we reduced the DNA origami concentration slightly from 3 nM to 2 nM to save material. However, since the further application of the assembled lattices in molecular lithography will most likely involve the washing and possibly drying of the sample,[^5^, ^21^] we also aimed at minimizing any negative influence on lattice homogeneity and regularity resulting from these processing steps. To this end, we incubated the sample after lattice assembly for another hour in 10 mM Ni^2+^‐containing buffer. Because of their high binding affinities, Ni^2+^ ions can replace both Mg^2+^ and Na^+^ from the mica‐DNA interface and thereby firmly attach the DNA origami nanostructures at their lattice positions to the mica surface.[^18^, ^21^]

**Figure 2:**
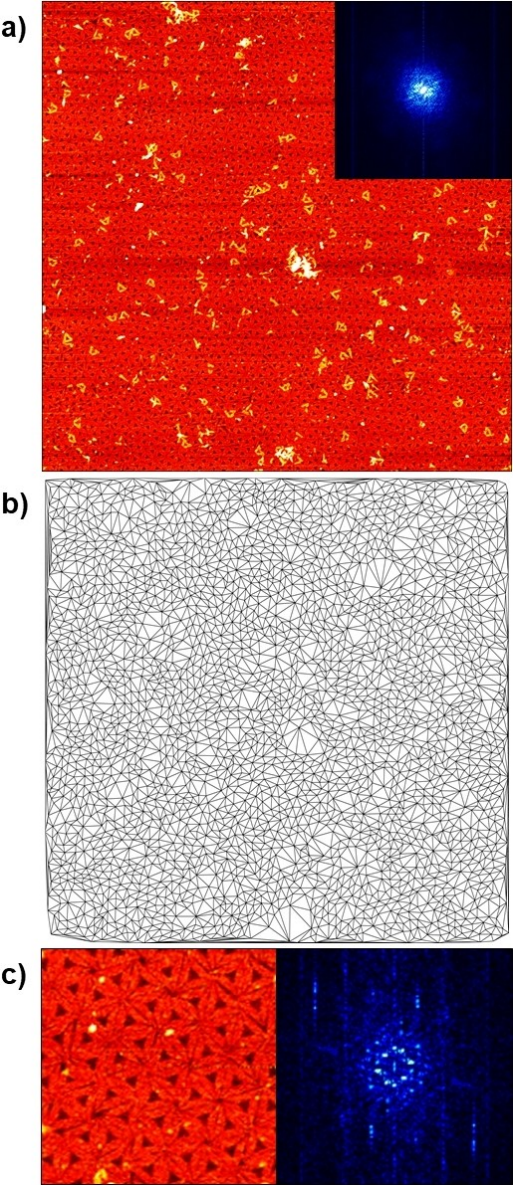
a) Representative AFM image (5×5 μm^2^) with corresponding 2D FFT (inset) of the DNA origami lattice assembled on the mica surface. b) Delaunay triangulation of the lattice shown in a). c) A 0.75×0.75 μm^2^ zoom of a single‐crystalline lattice grain (left) and the corresponding 2D FFT (right), respectively.

Figure 2a shows a 5×5 μm^2^ AFM image of the assembled DNA origami lattice recorded at a random location on the 7.5×2.5 cm^2^ mica surface. The polycrystalline microstructure of the lattice is apparent from the corresponding Delaunay triangulation depicted in Figure 2b. In particular, single‐crystalline domains with dimensions of several hundred nanometers can be observed that are separated by defect‐rich grain boundaries (see Figure 2a,b). These defects can be zero‐, one‐, and two‐dimensional and occur upon incorporation of partially folded DNA origami monomers from solution, when the boundaries of two growing crystallites come into contact, or during lattice rearrangements required to anneal existing defects or accommodate incoming monomers.^[20]^ Furthermore, DNA origami triangles are adsorbed on top of the assembled lattice. Such random deposition is often observed in the dry state,[^5^, ^16^] while *in‐situ* AFM images recorded in liquid show no indications of multilayer build‐up under otherwise comparable conditions.[^20^, ^23^, ^24^] We thus believe that these artefacts occur during sample washing when a meniscus moving across the surface randomly deposits residual DNA origami nanostructures from solution.^[14]^ In combination with scan artefacts, which cannot completely be avoided at such a large scan sizes, these features result in a rather poorly defined 2D Fast Fourier Transform (FFT). As shown in the inset in Figure 2a, the whole FFT is very noisy and does not display the intricate and complex symmetry obtained from smaller AFM images recorded under liquid conditions.[^20^, ^23^] Only for zooms of individual single‐crystalline grains such as the one shown in Figure 2c, rather well‐defined FFTs with clearly identifiable hexagonal symmetry can be obtained, which nevertheless suffer from drying and scanning artefacts.

To obtain a more reliable measure of lattice quality, we thus followed the more direct approach of topological pattern analysis, which is less affected by the artefacts mentioned above. Applying a previously established method,^[23]^ the coordinates of the centers of the DNA origami triangles in the lattice were determined and further used to derive a Delaunay triangulation of the lattice (see Figure 2b). From this triangulation, we have calculated two topological parameters (see Figure S2), namely the variance of the distribution of nearest neighbors (*μ*_2_) and the relative proportion of DNA origami triangles with exactly 6 neighbors (*p*_6_). According to Lemaitre *et al*.,^[26]^ these two parameters are related by

(1) $$\mu_{2}={\left(2\pi p_{6}^{2}\right)}^{-1},\;\mathrm{if}\;p_{6}<0.7\;\left(\mathrm{solid}\;\mathrm{line}\;\mathrm{in}\;\mathrm{Figure}\;3a\right)$$

(2) $$\mu_{2}=1-p_{6},\;\mathrm{if}\;p_{6}>0.7\;\left(\mathrm{dashed}\;\mathrm{line}\;\mathrm{in}\;\mathrm{Figure}\;3a\right)$$

Using this approach, we can quantify lattice order, packing, and homogeneity in a statistical manner over length scales accessible by AFM, *i. e*., 25 μm^2^ in the present case. Over such micrometer length scales, *μ*_2_ and *p*_6_ will mostly be governed by intrinsic growth kinetics, *i. e*., the size of the crystallites, and defect dynamics.^[20]^ Over macroscopic centimeter length scales, however, external processing conditions may result in additional inhomogeneities, for instance due to variations in the local concentration, liquid volume, or temperature, as well as from inhomogeneous washing and drying.^[16]^

**Figure 3:**
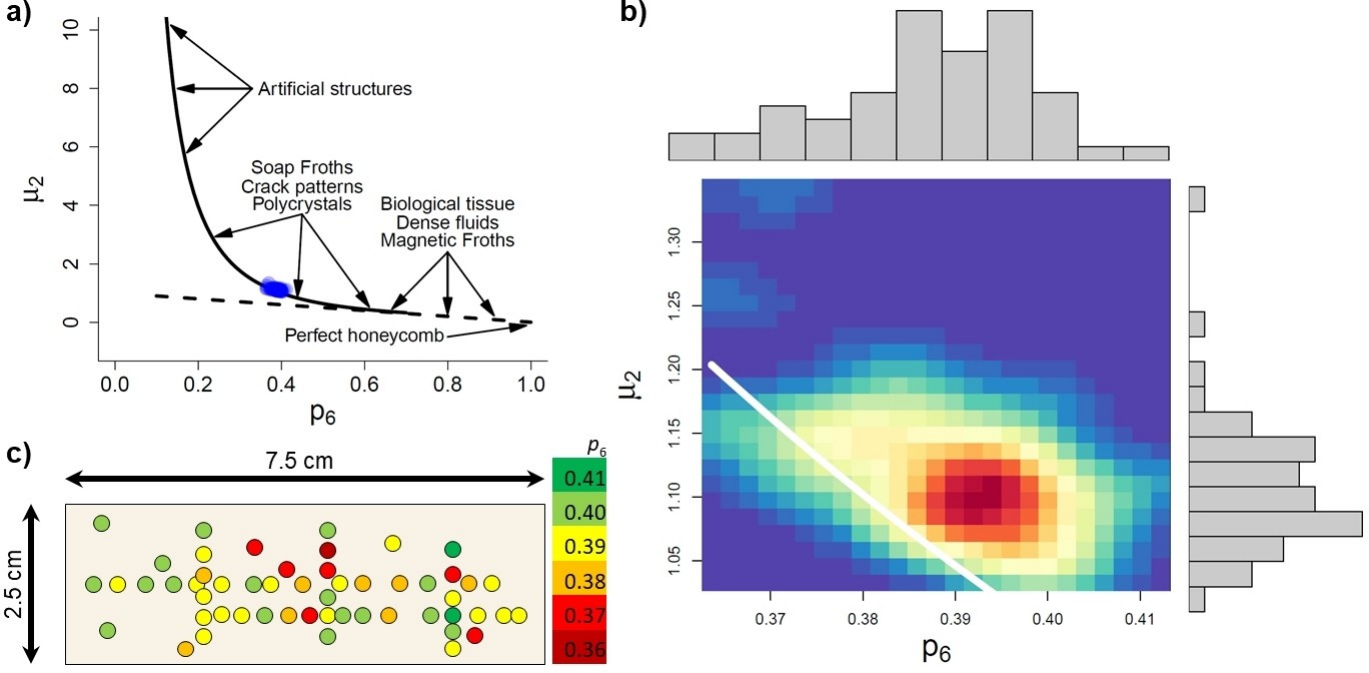
a) Lemaitre's plot for all the data to show the exact location of the experiment (blue dots). The solid and dashed curves follow Eqs. (1) and (2), respectively. For comparison, the regions of location of other natural and artificial patterns are indicated in the plot. b) Same as panel a) but zoomed and aggregated as a heat map. c) Heat map of the mica surface, linking the obtained *p*_6_ values to the locations of the corresponding AFM images.

In Figure 3a, we show the Lemaitre plot of the data points calculated from 53 individual AFM images recorded at different locations on the 7.5×2.5 cm^2^ mica surface (blue). The fact that the points do not fall onto the straight dashed line (Figure 3a) means that the packing is not perfect, as a purely hexagonal packing would shift the points towards *p*_6_→1 and *μ*_2_→0. As can be seen in the zoom in Figure 3b, the data points also do not fall exactly on top of the solid line. This is due to the errors made in the calculation for the points at the boundaries of the AFM images (see Figure 2b). Nevertheless, the obtained values lie right within the range expected for polycrystals. A remarkable feature of the data shown in Figure 3a,b concerns the variation between the individual data points, which is surprisingly small and thus indicates that the corresponding locations on the mica surface all share almost the same degree of order.

Quantitatively, the obtained *p*_6_ values range from 0.36 to 0.42 and are thus in perfect agreement with the ones determined *in situ* for smaller substrates under equivalent conditions.^[23]^ This indicates that the DNA origami triangles randomly deposited on the top of the formed lattice during washing and drying do not contribute significantly to this topological analysis. Furthermore, other self‐organized nanopatterns such as ion bombardment‐induced nanofoams and nanohole patterns on silicon surfaces typically show lower *p*_6_ values in the range from ∼0.2 to ∼0.3,^[27]^ which means that those systems display a lower degree of order than our lattice.

In order to develop a better understanding of the variation of lattice order over the entire mica surface, we have created a heat map that links the obtained *p*_6_ values to the locations of the corresponding AFM images. As can be seen in Figure 3c, the distribution of the *p*_6_ values over the entire surface area appears to be rather random and does not exhibit any systematic variations or trends such as boundary or edge effects. This is proof of the high homogeneity of the self‐assembled DNA origami lattice, which displays essentially the same degree of order over the entire 18.75 cm^2^ mica surface. In other words, order extends to areas containing hundreds of billions of DNA origami triangles.

**Figure 4:**
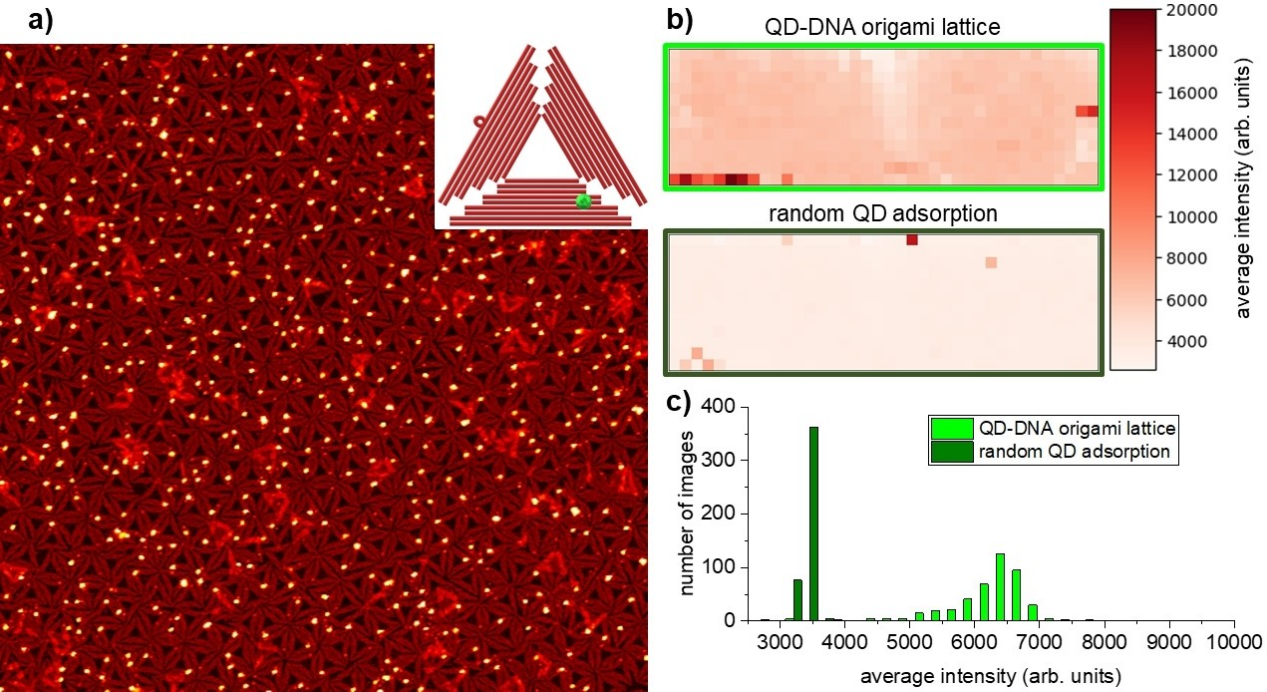
a) AFM image (2.5×2.5 μm^2^) of the QD‐decorated DNA origami lattice. The inset shows a scheme of the DNA origami triangle with a bound QD. b) Heat maps of the QD‐decorated DNA origami lattice depicted in the AFM image in a) and a DNA‐free control sample after random QD adsorption showing the lateral variation of the QD fluorescence intensities over almost the entire 7.5×2.5 cm^2^ surfaces (∼7×2 cm^2^ area imaged as the areas close to the edges of the mica substrate were neglected). c) Histograms of the recorded fluorescence intensities for the samples shown in b). For each sample, 456 fluorescence microscopy images have been analyzed.

However, AFM only provides snapshots of rather small surface areas with dimensions of a few micrometers and thus does not allow the entire sample surface to be mapped. Therefore, we have next turned to fluorescence microscopy to evaluate the homogeneity of the assembled DNA origami lattice over the full 18.75 cm^2^. To this end, another lattice was assembled at a mica substrate of the same size using the same protocol as before but DNA origami triangles featuring two biotinylated staple strands. The assembled DNA origami lattice was then exposed to streptavidin‐coated QDs. Because of the small distance between the neighboring biotin modifications, each DNA origami could bind just one QD (see Figure S1 and the inset in Figure 4a). As can be seen in the AFM image in Figure 4a, the QDs are indeed preferentially located on top of the DNA origami lattice. The QD coverage is rather homogeneous over micrometer length scales and an average QD binding yield of 52.8±3.6 % was determined based on 580 individual DNA origami triangles analyzed in six AFM images that were recorded at six random positions on the sample surface. This comparatively low binding yield can be attributed to the fact that the DNA origami triangles can adsorb face‐up or face‐down, while only one face displays the biotin modifications. The T_4_ spacers apparently are too short to enable the efficient threading of the buried biotin modifications through the DNA origami triangle,^[28]^ so that the QDs can only bind to those triangles, which are adsorbed with their biotin modifications pointing upward.

The fluorescence of the DNA origami‐bound QDs was then mapped by recording 456 images 394×291 μm^2^ in size (1500 μm spacing between neighboring images). Applying the average fluorescence intensity of each image, a heat map covering the entire sample surface was generated. As can be seen in Figure 4b, the fluorescence intensity of the QD‐decorated DNA origami lattice is rather homogeneously distributed over the full 18.75 cm^2^ surface. The only apparent inhomogeneities are a streak of lower intensity in the center and two high‐intensity regions right at the edges of the sample. We attribute both inhomogeneities to the difficulties experienced when manually handling such large samples, in particular during washing steps. For instance, the meniscus‐mediated random deposition of residual DNA origami triangles from solution on top of the assembled lattice during washing (see above) can be expected to be enhanced at the sample edges where droplets are easily trapped. Since these DNA origami also carry biotin modification, enhanced QD binding will occur in the corresponding areas. On the other hand, non‐specific QD deposition may also be enhanced close to the sample edges by the same effect.

We have also mapped an identically treated control sample without DNA origami lattice that showed only random QD adsorption. Here, the fluorescence intensities, in general, are much lower than for the sample with DNA origami lattice (see Figure 4b). This is particularly obvious when comparing the histograms of the fluorescence intensities displayed in Figure 4c and emphasizes that the QDs were indeed specifically binding to the biotin modification on the DNA origami triangles instead of adsorbing non‐specifically at the mica surface. Interestingly, the control sample also showed some high‐intensity spots at its edges, which further hints at inhomogeneous sample washing resulting in non‐specific QD deposition at the sample edges.

## Conclusion

In summary, we have demonstrated the surface‐assisted assembly of a polycrystalline DNA origami lattice over a macroscopic surface area of 18.75 cm^2^. The topological analysis of more than 50 AFM images recorded at random locations all over the sample surface showed only minuscule and random variations in the quality and order of the assembled lattice. The homogeneity of DNA origami lattice assembly over the entire surface area was assessed by imaging more than 450 locations/sample with fluorescence microscopy, using biotin‐modified DNA origami nanostructures for the selective binding of streptavidin‐coated QDs. While the results of these measurements revealed a very homogeneous surface coverage over cm^2^ areas, some minor inhomogeneities were nevertheless observed, mostly at the sample edges. These inhomogeneities probably result from the difficulties in the manual handling of such comparatively large samples, in particular during sample washing. Such problems may be overcome in the future by employing automated sample handling systems such as dip coaters.

In order to assemble DNA origami lattices with hexagonal symmetry over macroscopic length scales, we employed a well‐established protocol previously optimized for smaller surface areas and simply included an additional fixation step to minimize washing and drying artefacts. Since the employed protocol is rather similar to other protocols that have been used for the assembly of various DNA origami nanostructures into lattices with different geometries,[^18^, ^22^, ^24^] we believe that lattices of similar size can be obtained with this approach also from various other DNA origami shapes. Furthermore, the applied protocol should also be compatible with the crosslinking of the individual DNA origami nanostructures in the lattice via attractive interactions such as blunt‐end‐stacking^[18]^ or sticky‐end hybridization.^[19]^ This may be exploited in future implementations of this approach and in particular enable the transfer of the assembled DNA origami lattices from mica to technologically more relevant substrates such as silicon.[^22^, ^23^]

For the fabrication of an 18.75 cm^2^ DNA origami lattice, we used in total 2 pmol DNA origami triangles, including excess DNA origami nanostructures that were washed away after lattice assembly. At a current price of € 0.794 per pmol, this amounts to € 1.59 for the M13mp18 scaffold. The Rothemund triangle contains in total 7194 staple nt and folding was performed at 10‐fold excess of staples to scaffold. Therefore, a total 143.88 nmol staple nt was required for the fabrication of the DNA origami lattice, which at an average rate of € 0.005 per nmol nt amounts to € 0.72. The total costs for the synthetic and genomic DNA required for the fabrication of an 18.75 cm^2^ DNA origami lattice hence amount to about € 2.31, corresponding to € 0.12 per cm^2^. Remarkably, this value is comparable to the costs of binary reticles employed in photolithography, which contribute $ 0.003–$ 0.61 per cm^2^ to each wafer exposure.^[29]^ Our work thus demonstrates not only the possibility of fabricating homogeneous DNA origami lattices over macroscopic surface areas of several cm^2^, but also its economic feasibility. The surface‐assisted assembly of DNA origami lattices therefore represents a promising approach to large‐scale surface patterning with many potential applications in materials science and surface engineering.

## Experimental Section

**DNA origami folding and lattice assembly**: Rothemund triangles^[3]^ were folded as described previously^[24]^ by folding the 7,249 nt M13mp18 scaffold (Tilibit) in 1× TAE buffer (Carl Roth) containing 10 mM MgCl_2_ (Sigma‐Aldrich) via hybridization with 208 staple strands (Metabion). The folded DNA origami triangles were purified by spin filtering using Amicon Ultra filters with 100 kDa MWCO (Millipore) and the resulting DNA origami concentration determined using an IMPLEN NanoPhotometer P 330. This assembly protocol typically yields ≥90 % of correctly folded and structurally intact DNA origami triangles as determined by AFM.^[30]^

For a 7.5 cm×2.5 cm DNA origami lattice, 1 ml solution was used containing 2 nM DNA origami triangles in 1× TAE buffer with 10 mM MgCl_2_ and 75 mM NaCl (Sigma‐Aldrich). The solution was gently and evenly deposited onto the freshly cleaved mica surface (grade V1, Ted Pella) and incubated for 2 h. After incubation, the mica surface was rinsed several times with 1× TAE buffer containing 10 mM MgCl_2_. Immediately after rinsing, 1× TAE buffer containing 10 mM NiCl_2_ (Sigma‐Aldrich) was deposited on the mica surface. After incubation for 1 h, the surface was rinsed several times with HPLC‐grade water (VWR) and blow‐dried in a stream of ultrapure air.

For the assembly of the quantum dot (QD)‐modified lattice, two neighboring staple strands in the DNA origami triangle were extended by single‐stranded T_4_ spacer sequences, which protruded from the DNA origami surface and displayed biotin modifications at their distal ends (see table S1 and Figure S1). After DNA origami lattice assembly, a 10 nM solution of streptavidin‐coated QDs (QDot™ 605 streptavidin conjugate, Thermo Scientific) in 1× TAE buffer containing 10 mM MgCl_2_ was deposited on the sample surface. After incubation for 2 h in the dark, the surface was rinsed with HPLC‐grade water and blow‐dried in a stream of ultrapure air.

**AFM imaging and image analysis**: The DNA origami lattices were imaged in air by AFM (Dimension Icon, Bruker) in ScanAsyst mode using ScanAsyst‐Air cantilevers (Bruker). AFM images were recorded with scan sizes of 5×5 μm^2^ with a resolution of 1024 px×1024 px. The AFM images were preprocessed in Gwyddion^[31]^ using mean plane subtraction and row alignment by a 1^st^ degree polynomial. The color range was set to automatic with tails cut off. Automated analysis of the preprocessed AFM images was performed as described previously.^[23]^

**Fluorescence microscopy**: The QD‐decorated DNA origami lattice and DNA‐free control sample were characterized by a fluorescence microplate reader (Cytation 3 Cell Imaging Multi‐Mode Reader, BioTek Instruments) equipped with a 20× objective. The fluorescence micrographs were acquired using an excitation wavelength of 586 nm and emission wavelength of 647 nm. The size of each image was 394×291 μm^2^ with a resolution of 1224 px×904 px. Both the vertical and horizontal spacings between the neighboring images were 1500 μm (large‐scale maps of the imaging locations are shown in Figure S3). The average intensity of each image was calculated as a mean value of all pixels.

## Conflict of interest

The authors declare no conflict of interest.

## Supporting information

As a service to our authors and readers, this journal provides supporting information supplied by the authors. Such materials are peer reviewed and may be re‐organized for online delivery, but are not copy‐edited or typeset. Technical support issues arising from supporting information (other than missing files) should be addressed to the authors.

Supplementary

## References

[1a] E. Braun , Y. Eichen , U. Sivan , G. Ben-Yoseph , Nature 1998, 391, 775; 9486645 10.1038/35826

[1b] C. F. Monson , A. T. Woolley , Nano Lett. 2003, 3, 359;

[1c] H. Yan , S. H. Park , G. Finkelstein , J. H. Reif , T. H. LaBean , Science 2003, 301, 1882; 14512621 10.1126/science.1089389

[1d] J. Richter , R. Seidel , R. Kirsch , M. Mertig , W. Pompe , J. Plaschke , H. K. Schackert , Adv. Mater. 2000, 12, 507.

[2a] H. A. Becerril , A. T. Woolley , Chem. Soc. Rev. 2009, 38, 329; 19169451 10.1039/b718440a

[2b] L. Hui , Q. Zhang , W. Deng , H. Liu , Small 2019, 15, e1805428; 30811832 10.1002/smll.201805428

[2c] R. Wang , G. Zhang , H. Liu , Curr. Opin. Colloid Interface Sci. 2018, 38, 88; 10.1016/j.cocis.2018.10.009 PMC6615560 31289450

[2d] A. Xu , J. N. Harb , M. A. Kostiainen , W. L. Hughes , A. T. Woolley , H. Liu , A. Gopinath , MRS Bull. 2017, 42, 943.

[3] P. W. K. Rothemund , Nature 2006, 440, 297. 16541064 10.1038/nature04586

[4a] H. Kim , K. Arbutina , A. Xu , H. Liu , Beilstein J. Nanotechnol. 2017, 8, 2363; 29181293 10.3762/bjnano.8.236 PMC5687006

[4b] S. P. Surwade , F. Zhou , Z. Li , A. Powell , C. O′Donnell , H. Liu , Chem. Commun. 2016, 52, 1677; 10.1039/c5cc08183a 26661791

[4c] C. Tian , H. Kim , W. Sun , Y. Kim , P. Yin , H. Liu , ACS Nano 2017, 11, 227; 28052196 10.1021/acsnano.6b04777

[4d] M. Sajfutdinow , K. Uhlig , A. Prager , C. Schneider , B. Abel , D. M. Smith , Nanoscale 2017, 9, 15098; 28967945 10.1039/c7nr03696e

[4e] H. Aslan , A. Krissanaprasit , F. Besenbacher , K. V. Gothelf , M. Dong , Nanoscale 2016, 8, 15233; 27487933 10.1039/c6nr03199d

[4f] K. Busuttil , A. Rotaru , M. Dong , F. Besenbacher , K. V. Gothelf , Chem. Commun. 2013, 49, 1927; 10.1039/c3cc37408d 23361330

[4g] Y. Tokura , S. Harvey , X. Xu , C. Chen , S. Morsbach , K. Wunderlich , G. Fytas , Y. Wu , D. Y. W. Ng , T. Weil , Chem. Commun. 2018, 54, 2808. 10.1039/c7cc09620h PMC5885267 29492501

[5] S. Ramakrishnan , S. Subramaniam , A. F. Stewart , G. Grundmeier , A. Keller , ACS Appl. Mater. Interfaces 2016, 8, 31239. 27779405 10.1021/acsami.6b10535

[6a] S. P. Surwade , F. Zhou , B. Wei , W. Sun , A. Powell , C. O′Donnell , P. Yin , H. Liu , J. Am. Chem. Soc. 2013, 135, 6778; 23574340 10.1021/ja401785h

[6b] S. P. Surwade , S. Zhao , H. Liu , J. Am. Chem. Soc. 2011, 133, 11868; 21751796 10.1021/ja2038886

[6c] B. Shen , V. Linko , K. Tapio , M. A. Kostiainen , J. J. Toppari , Nanoscale 2015, 7, 11267; 26066528 10.1039/c5nr02300a

[6d] X. Liu , X. Jing , P. Liu , M. Pan , Z. Liu , X. Dai , J. Lin , Q. Li , F. Wang , S. Yang , et al., Chem 2020, 6, 472;

[6e] X. Liu , F. Zhang , X. Jing , M. Pan , P. Liu , W. Li , B. Zhu , J. Li , H. Chen , L. Wang et al , Nature 2018, 559, 593; 30013119 10.1038/s41586-018-0332-7

[6f] L. Nguyen , M. Döblinger , T. Liedl , A. Heuer-Jungemann , Angew. Chem. Int. Ed. 2019, 58, 912; 10.1002/anie.201811323 30398705

[6g] B. Uprety , E. P. Gates , Y. Geng , A. T. Woolley , J. N. Harb , Langmuir 2014, 30, 1134; 24410066 10.1021/la403617r

[6h] B. Uprety , J. Jensen , B. R. Aryal , R. C. Davis , A. T. Woolley , J. N. Harb , Langmuir 2017, 33, 10143; 28876958 10.1021/acs.langmuir.7b01659

[6i] S. Helmi , C. Ziegler , D. J. Kauert , R. Seidel , Nano Lett. 2014, 14, 6693; 25275962 10.1021/nl503441v

[6j] L. Hui , R. Nixon , N. Tolman , J. Mukai , R. Bai , R. Wang , H. Liu , ACS Nano 2020, 14, 13047; 33048526 10.1021/acsnano.0c04493

[6k] M.-K. Nguyen , V. H. Nguyen , A. K. Natarajan , Y. Huang , J. Ryssy , B. Shen , A. Kuzyk , Chem. Mater. 2020, 32, 6657;

[6l] P. Piskunen , B. Shen , A. Keller , J. J. Toppari , M. A. Kostiainen , V. Linko , ACS Appl. Nano Mater. 2021, 4, 529.

[7] B. Shen , V. Linko , K. Tapio , S. Pikker , T. Lemma , A. Gopinath , K. V. Gothelf , M. A. Kostiainen , J. J. Toppari , Sci. Adv. 2018, 4, eaap8978. 29423446 10.1126/sciadv.aap8978 PMC5804581

[8] B. Uprety , T. Westover , M. Stoddard , K. Brinkerhoff , J. Jensen , R. C. Davis , A. T. Woolley , J. N. Harb , Langmuir 2017, 33, 726. 28075137 10.1021/acs.langmuir.6b04097

[9] B. R. Aryal , D. R. Ranasinghe , T. R. Westover , D. G. Calvopiña , R. C. Davis , J. N. Harb , A. T. Woolley , Nano Res. 2020, 557, 696.

[10] B. R. Aryal , T. R. Westover , D. R. Ranasinghe , D. G. Calvopiña , B. Uprety , J. N. Harb , R. C. Davis , A. T. Woolley , Langmuir 2018, 34, 15069. 30176148 10.1021/acs.langmuir.8b02225

[11] T. Bayrak , S. Helmi , J. Ye , D. Kauert , J. Kelling , T. Schönherr , R. Weichelt , A. Erbe , R. Seidel , Nano Lett. 2018, 18, 2116. 29482327 10.1021/acs.nanolett.8b00344

[12a] K. Brassat , S. Ramakrishnan , J. Bürger , M. Hanke , M. Doostdar , J. K. N. Lindner , G. Grundmeier , A. Keller , Langmuir 2018, 34, 14757; 29754490 10.1021/acs.langmuir.8b00793

[12b] B. Ding , H. Wu , W. Xu , Z. Zhao , Y. Liu , H. Yu , H. Yan , Nano Lett. 2010, 10, 5065; 21070012 10.1021/nl1033073 PMC3399060

[12c] B. Gao , K. Sarveswaran , G. H. Bernstein , M. Lieberman , Langmuir 2010, 26, 12680; 20590122 10.1021/la101343k

[12d] A. E. Gerdon , S. S. Oh , K. Hsieh , Y. Ke , H. Yan , H. T. Soh , Small 2009, 5, 1942; 19437465 10.1002/smll.200900442

[12e] A. Gopinath , P. W. K. Rothemund , ACS Nano 2014, 8, 12030; 25412345 10.1021/nn506014s

[12f] R. J. Kershner , L. D. Bozano , C. M. Micheel , A. M. Hung , A. R. Fornof , J. N. Cha , C. T. Rettner , M. Bersani , J. Frommer , P. W. K. Rothemund et al , Nat. Nanotechnol. 2009, 4, 557; 19734926 10.1038/nnano.2009.220

[12g] B. Teshome , S. Facsko , A. Keller , Nanoscale 2014, 6, 1790; 24352681 10.1039/c3nr04627c

[12h] A. C. Pearson , E. Pound , A. T. Woolley , M. R. Linford , J. N. Harb , R. C. Davis , Nano Lett. 2011, 11, 1981. 21473607 10.1021/nl200306w

[13] A. Kopielski , A. Csaki , W. Fritzsche , Langmuir 2015, 31, 12106. 26488367 10.1021/acs.langmuir.5b03137

[14] B. Teschome , S. Facsko , K. V. Gothelf , A. Keller , Langmuir 2015, 31, 12823. 26522180 10.1021/acs.langmuir.5b02569

[15a] A. P. Nievergelt , C. Kammer , C. Brillard , E. Kurisinkal , M. M. C. Bastings , A. Karimi , G. E. Fantner , Small Methods 2019, 3, 1900031;

[15b] Y. Sato , M. Endo , M. Morita , M. Takinoue , H. Sugiyama , S. Murata , S. M. Nomura , Y. Suzuki , Adv. Mater. Interfaces 2018, 17, 1800437;

[15c] Y. Suzuki , M. Endo , H. Sugiyama , Nat. Commun. 2015, 6, 8052; 26310995 10.1038/ncomms9052 PMC4560778

[15d] S. Kempter , A. Khmelinskaia , M. T. Strauss , P. Schwille , R. Jungmann , T. Liedl , W. Bae , ACS Nano 2019, 13, 996; 30588792 10.1021/acsnano.8b04631

[15e] S. Hamada , S. Murata , Angew. Chem. Int. Ed. 2009, 48, 6820; 10.1002/anie.200902662 19688799

[15f] N. Avakyan , J. W. Conway , H. F. Sleiman , J. Am. Chem. Soc. 2017, 139, 12027. 28783358 10.1021/jacs.7b06572

[16] S. Ramakrishnan , G. Grundmeier , A. Keller , Meth. Mol. Biol. 2018, 1811, 253. 10.1007/978-1-4939-8582-1_17 29926458

[17] Y. Suzuki , H. Sugiyama , M. Endo , Angew. Chem. Int. Ed. 2018, 57, 7061; 10.1002/anie.201801983 29644771

[18] S. Woo , P. W. K. Rothemund , Nat. Commun. 2014, 5, 4889. 25205175 10.1038/ncomms5889

[19] S. Kocabey , S. Kempter , J. List , Y. Xing , W. Bae , D. Schiffels , W. M. Shih , F. C. Simmel , T. Liedl , ACS Nano 2015, 9, 3530. 25734977 10.1021/acsnano.5b00161 PMC4415451

[20] C. Kielar , S. Ramakrishnan , S. Fricke , G. Grundmeier , A. Keller , ACS Appl. Mater. Interfaces 2018, 10, 44844. 30501167 10.1021/acsami.8b16047

[21] L. Liu , M. Zheng , Z. Li , Q. Li , C. Mao , ACS Appl. Mater. Interfaces 2019, 11, 13853. 30793605 10.1021/acsami.8b22691

[22] A. Aghebat Rafat , T. Pirzer , M. B. Scheible , A. Kostina , F. C. Simmel , Angew. Chem. Int. Ed. 2014, 53, 7665; 10.1002/anie.201403965 24894973

[23] Y. Xin , S. Martinez Rivadeneira , G. Grundmeier , M. Castro , A. Keller , Nano Res. 2020, 13, 3142.

[24] Y. Xin , X. Ji , G. Grundmeier , A. Keller , Nanoscale 2020, 12, 9733. 32324191 10.1039/d0nr01252a

[25a] J. J. Richardson , M. Björnmalm , F. Caruso , Science 2015, 348, aaa2491; 25908826 10.1126/science.aaa2491

[25b] J. J. Richardson , J. Cui , M. Björnmalm , J. A. Braunger , H. Ejima , F. Caruso , Chem. Rev. 2016, 116, 14828. 27960272 10.1021/acs.chemrev.6b00627

[26a] J. Lemaítre , A. Gervois , J. P. Troadec , N. Rivier , M. Ammi , L. Oger , D. Bideau , Philos. Mag. B 1993, 67, 347;

[26b] A. Gervois , J. P. Troadec , J. Lemaitre , J. Phys. A: Math. Gen. 1992, 25, 6169.

[27a] M. Castro , R. Cuerno , M. M. García-Hernández , L. Vázquez , Phys. Rev. Lett. 2014, 112, 94103; 10.1103/PhysRevLett.112.094103 24655256

[27b] A. Redondo-Cubero , K. Lorenz , F. J. Palomares , A. Muñoz , M. Castro , J. Muñoz-García , R. Cuerno , L. Vázquez , J. Phys. Condens. Matter 2018, 30, 274001. 29794326 10.1088/1361-648X/aac79a

[28] N. Wu , D. M. Czajkowsky , J. Zhang , J. Qu , M. Ye , D. Zeng , X. Zhou , J. Hu , Z. Shao , B. Li et al , J. Am. Chem. Soc. 2013, 135, 12172. 23924191 10.1021/ja403863a

[29] H. J. Levinson, *Principles of Lithography, Third Edition*, SPIE, Bellingham, WA, USA, **2010**.

[30] Y. Xin , C. Kielar , S. Zhu , C. Sikeler , X. Xu , C. Möser , G. Grundmeier , T. Liedl , A. Heuer-Jungemann , D. M. Smith , et al., Small 2020, 16, 1905959. 10.1002/smll.201905959 32130783

[31] D. Nečas , P. Klapetek , Open Phys. 2012, 10, 99.

