# Supplementary material for: Scaling Up DNA Origami Lattice Assembly
Source: Chemistry. 2021 May 4;27(33):8564–71. doi: 10.1002/chem.202100784 (PMC8252642; doi:10.1002/chem.202100784)
Supplement: Supplementary file 1 — Supplementary [file CHEM-27-8564-s001.pdf]

// Chemistry–A European Journal

Supporting Information

Scaling Up DNA Origami Lattice Assembly

Yang Xin, Boxuan Shen, Mauri A. Kostiainen, Guido Grundmeier, Mario Castro, Veikko Linko,* and Adrian Keller*

Author Contributions

Y.X. Conceptualization:Supporting; Data curation:Equal; Formal analysis:Equal; Investigation:Equal; Methodology:Equal; Validation:Equal; Visualization:Equal; Writing – original draft:Supporting; Writing – review & editing:Equal

B.S. Conceptualization:Supporting; Data curation:Equal; Formal analysis:Equal; Investigation:Equal; Methodology:Equal; Validation:Equal; Visualization:Equal; Writing – original draft:Supporting; Writing – review & editing:Equal

M.K. Supervision:Supporting; Writing – review & editing:Equal

G.G. Supervision:Supporting; Writing – review & editing:Equal

M.C. Data curation:Equal; Formal analysis:Lead; Methodology:Equal; Validation:Equal; Visualization:Equal; Writing – review & editing:Equal

V.L. Conceptualization:Lead; Methodology:Equal; Supervision:Supporting; Writing – original draft:Supporting; Writing – review & editing:Equal

A.K. Conceptualization:Lead; Data curation:Equal; Formal analysis:Equal; Methodology:Equal; Supervision:Lead; Validation:Equal; Visualization:Equal; Writing – original draft:Lead; Writing – review & editing:Equal

Sequences and locations of the biotin-modified staple strands

Table S1. Sequences of the biotin-modified staple strands. The biotin modification is indicated by Bt, the T4 spacers are highlighted in bold face. The staple ID corresponds to Rothemund's original notation.

staple ID	sequence (5'→ 3')
t6s5g	Bt-**TTTT**CAGAGCCAGGAGGTTGAGGCAGGTAACAGTGCCCG
t6s7f	ATTAAAGGCCGTAATCAGTAGCGAGCCACCCT**TTTT**-Bt

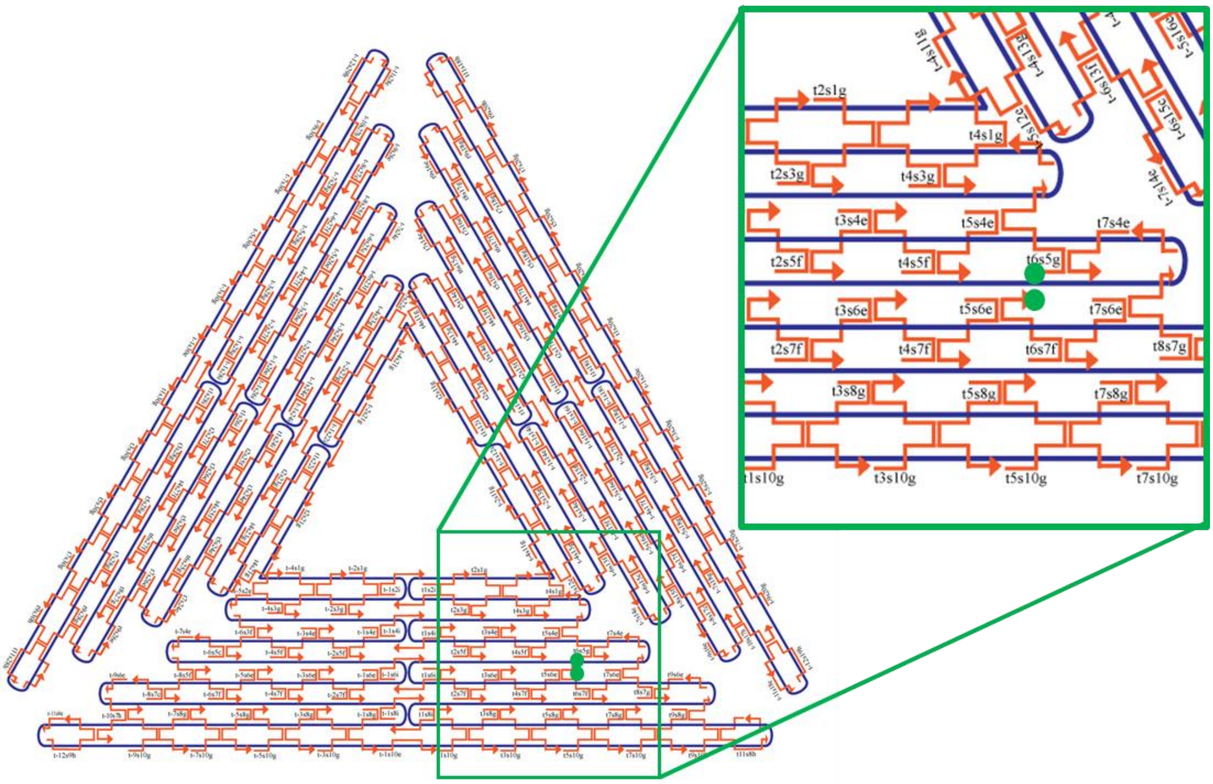

Figure S1. Map showing the staple (red) and scaffold (blue) arrangement in the DNA origami triangle. The locations of the biotin modifications used for QD binding are indicated by the green circles.

Definition of the topological parameters p_6 and μ_2

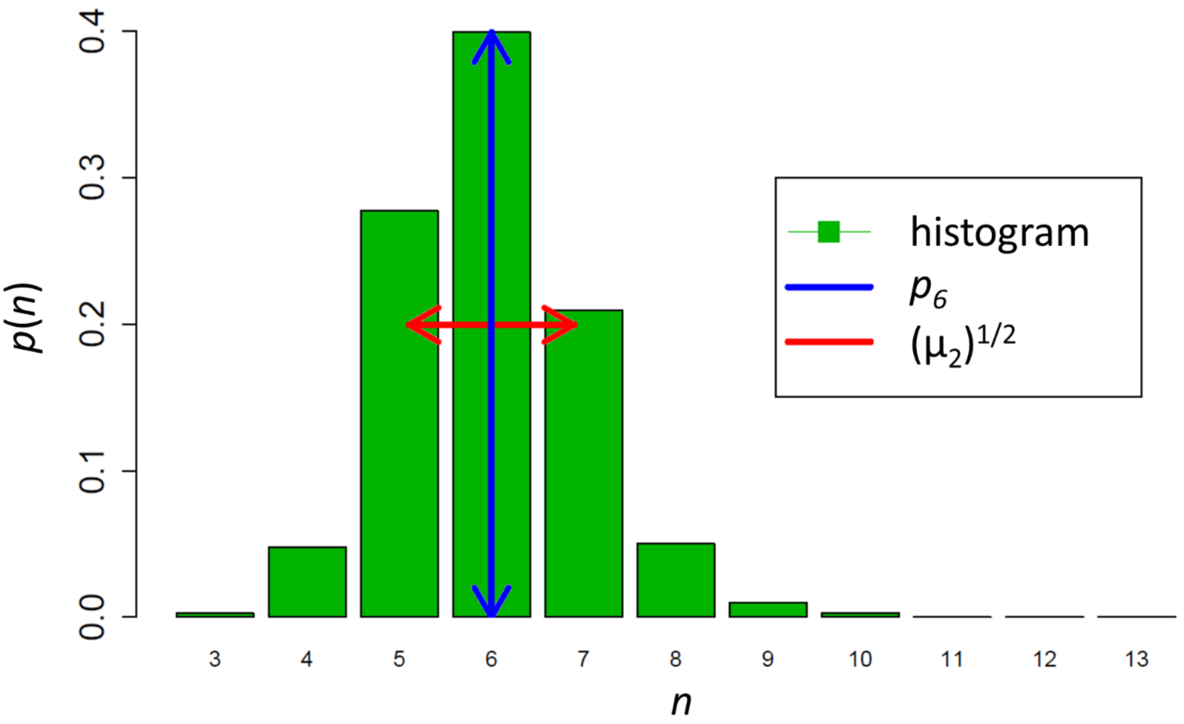

Figure S2. Example distribution of nearest neighbors for a DNA origami lattice. The topological parameters μ_2 and p_6 are indicated by the arrows.

Fluorescence microscopy investigation

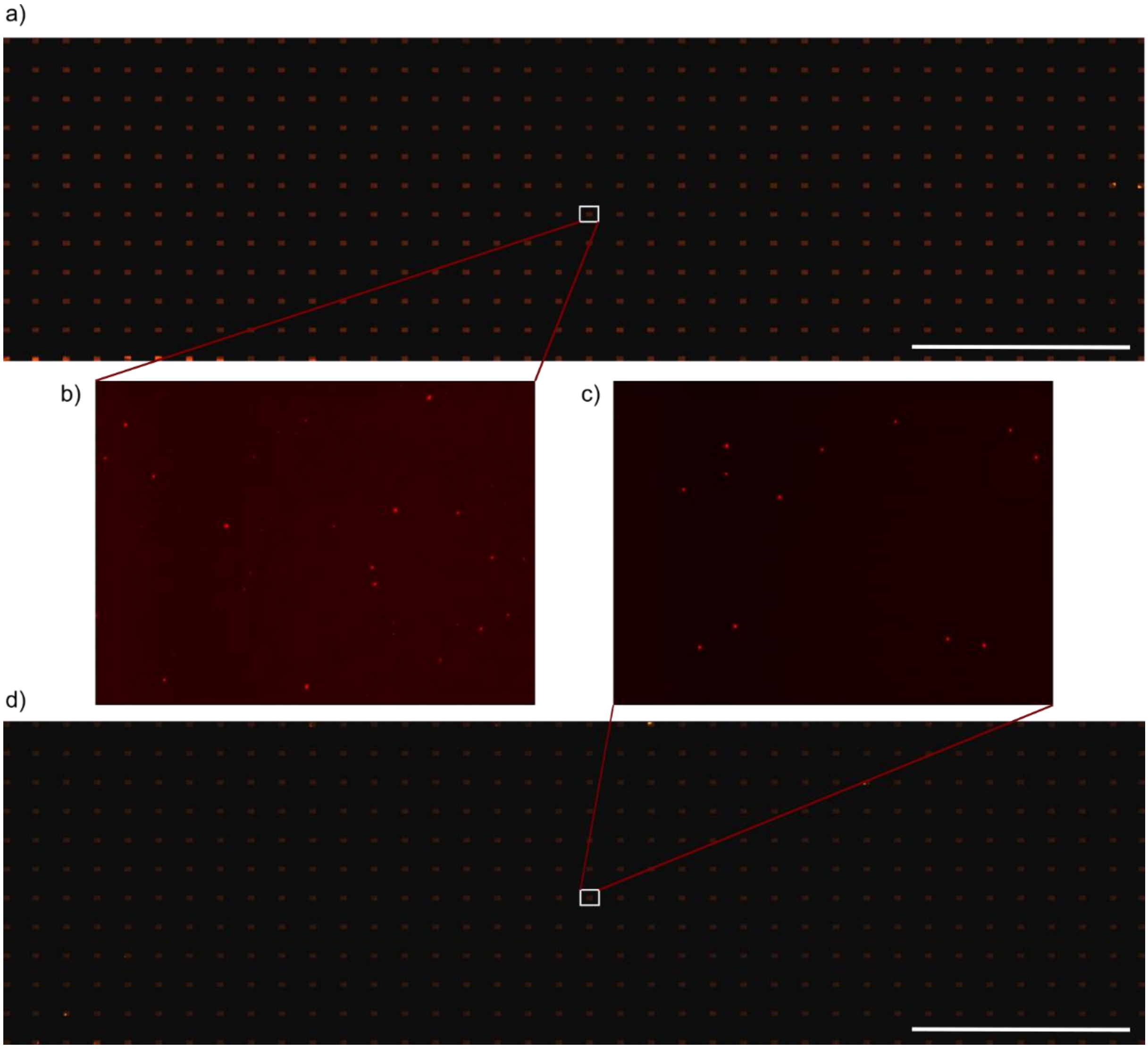

Figure S3. Fluorescence microscopy mapping and representative individual micrographs. a) and d) mapping of QD-decorated DNA origami lattice sample and DNA-free control sample, respectively. Scale bars: 1 cm. b) and c) representative individual images from the selected positions. Image sizes: 394 × 291 µm^2. Brightness and contrast of the map and individual images were adjusted with the same values to facilitate the visual comparison. DNA origami-bound QDs are responsible for the background intensity seen in the zoomed images (b,c), while the bright spots represent QD aggregates that are observed with and without DNA origami lattice. Integration was performed over the complete image and thus includes both dispersed and aggregated QDs.

All recorded AFM images and their corresponding p_6 and μ_2 values

The raw AFM image files can be found under: https://osf.io/7fvrd/

Image	p_6	μ_2
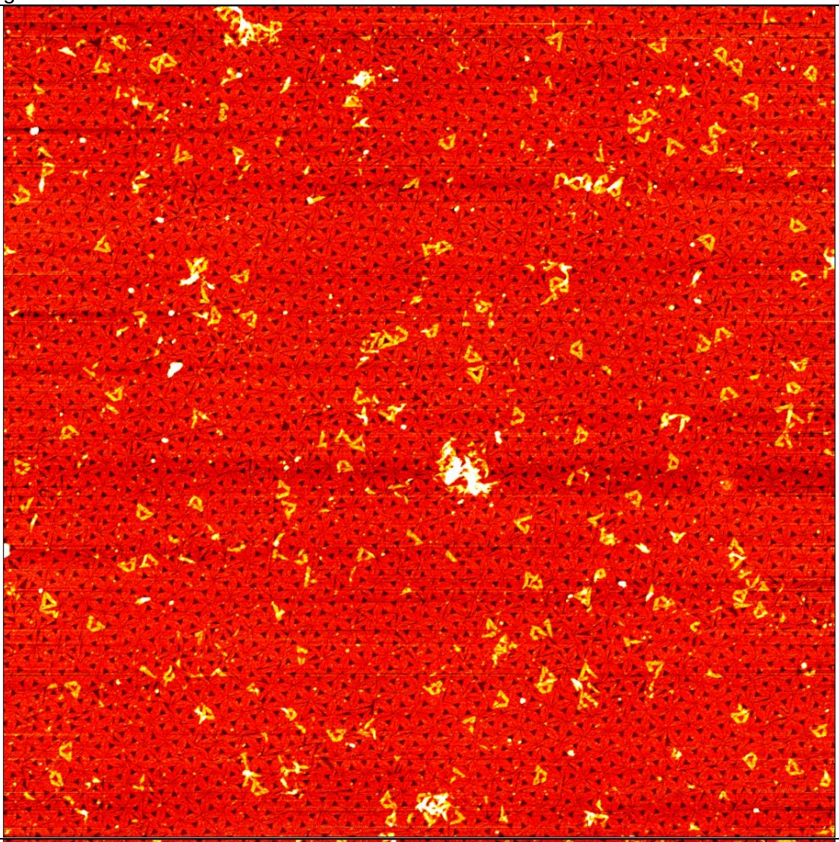	0.40	1.11
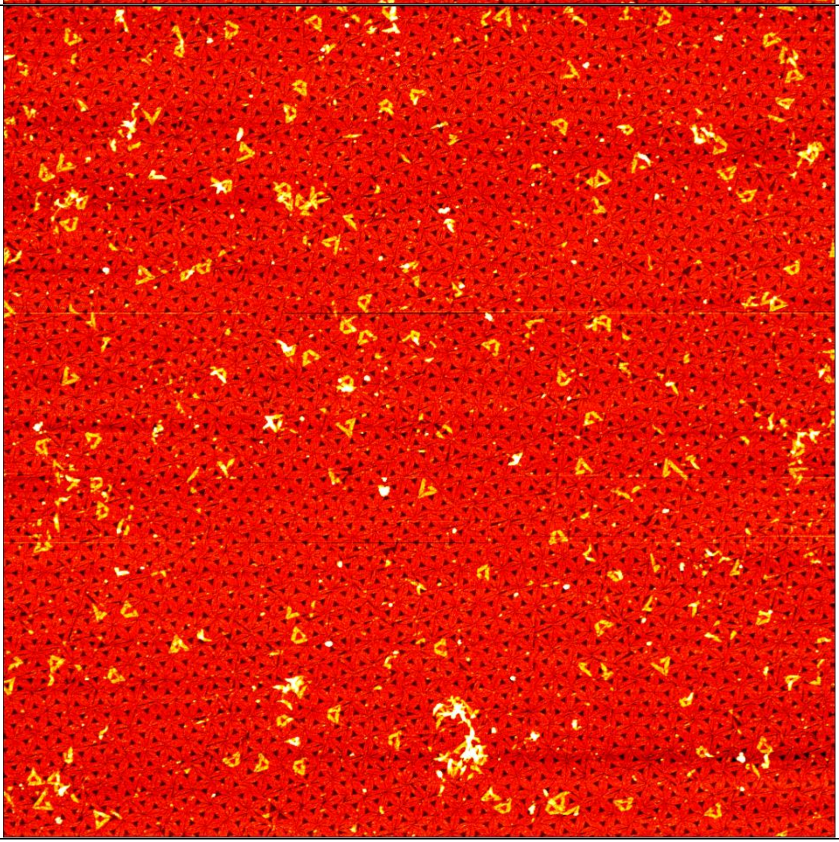	0.40	1.10

Image	p_6	μ_2
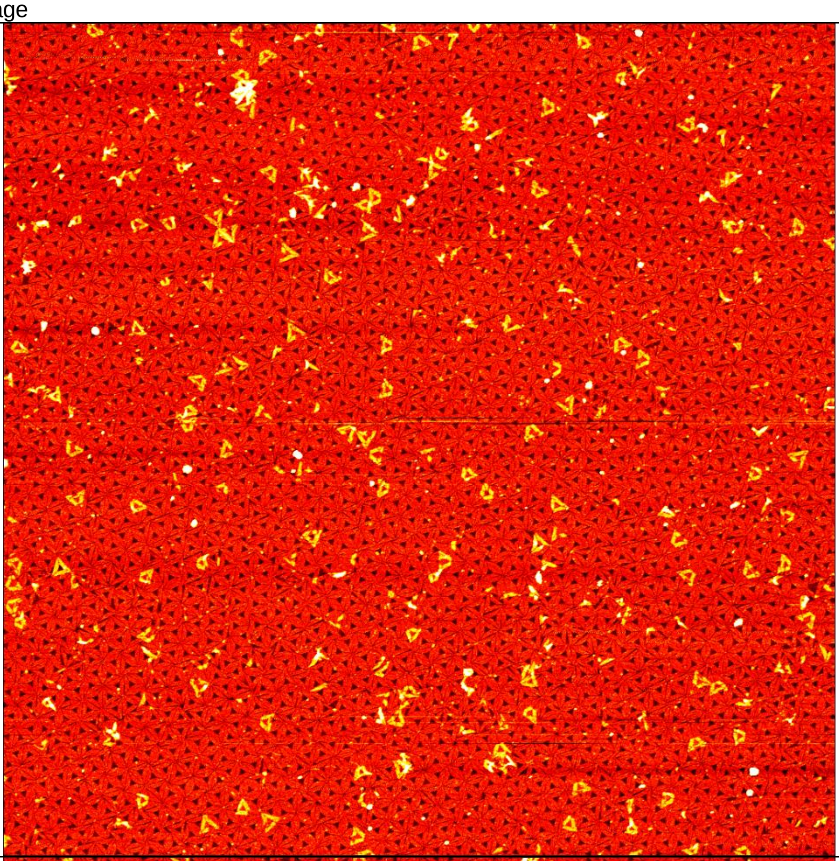	0.40	1.08
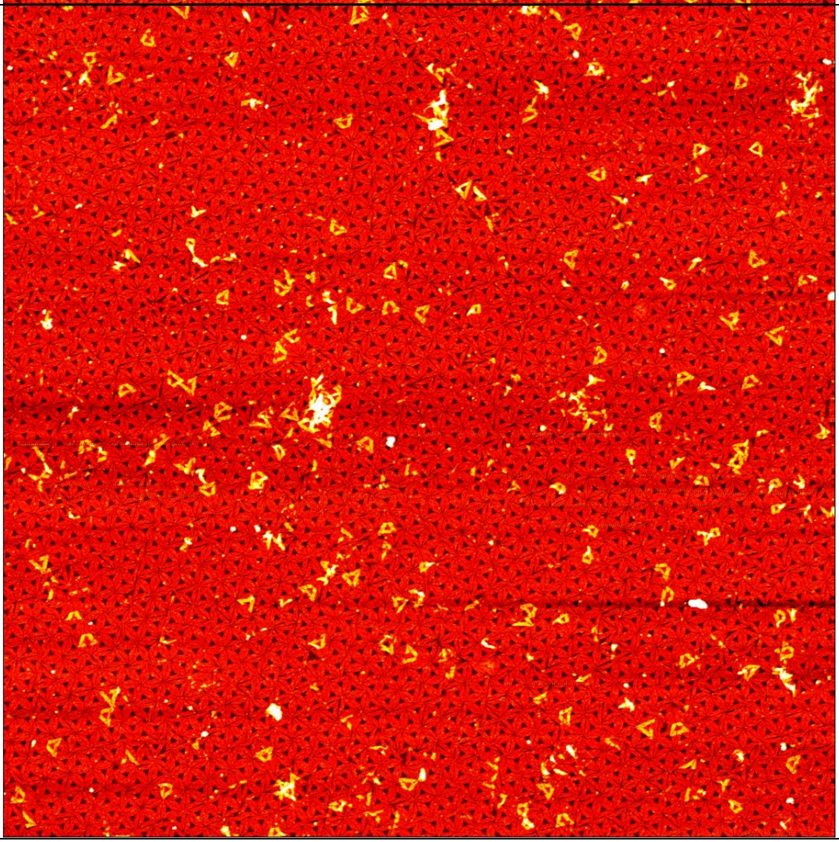	0.38	1.10

Image	p_6	μ_2
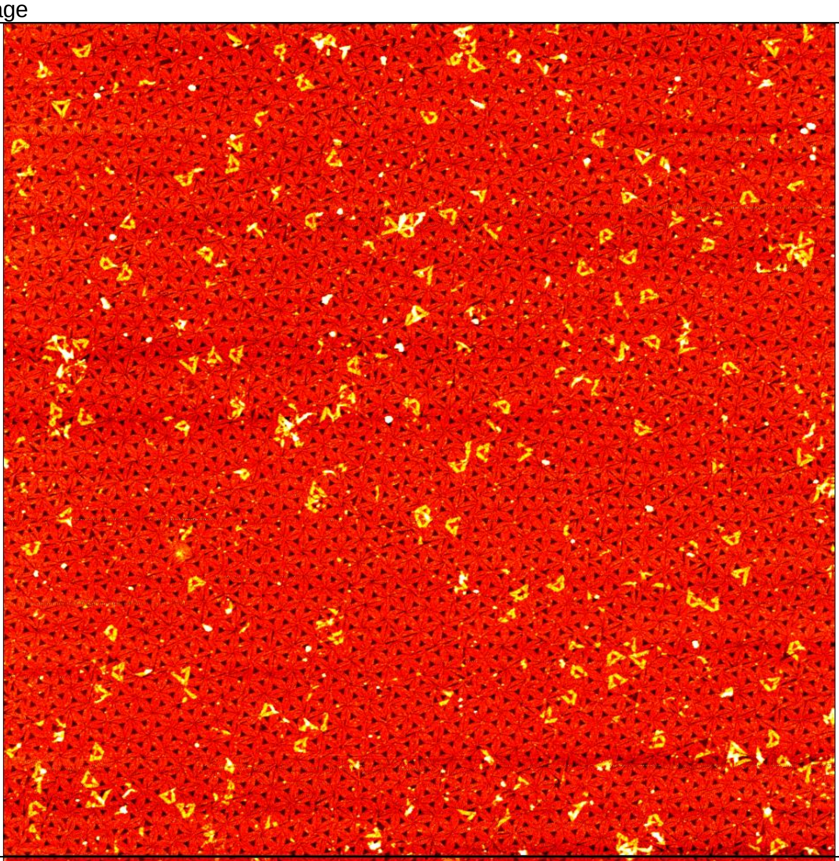	0.39	1.09
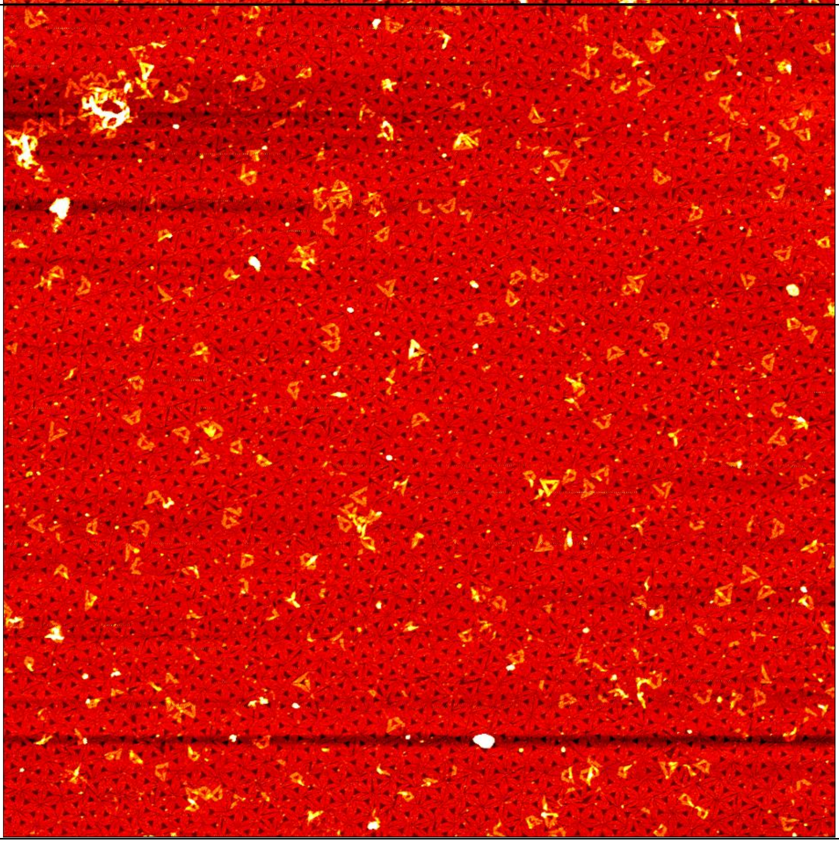	0.37	1.18

Image	p_6	μ_2
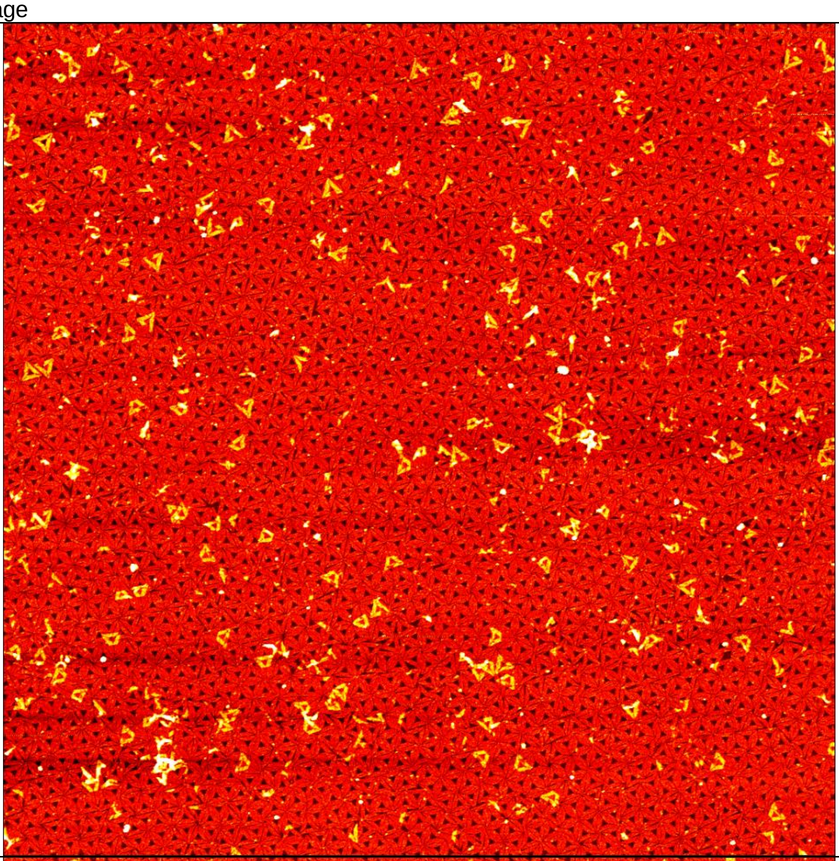	0.37	1.17
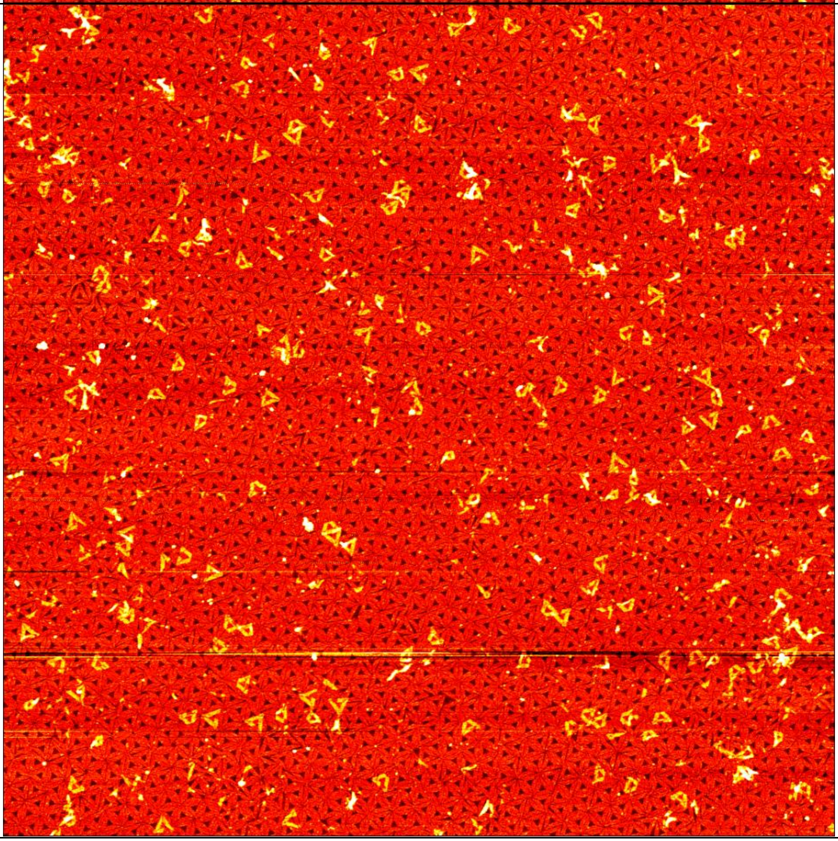	0.39	1.14

Image	p_6	μ_2
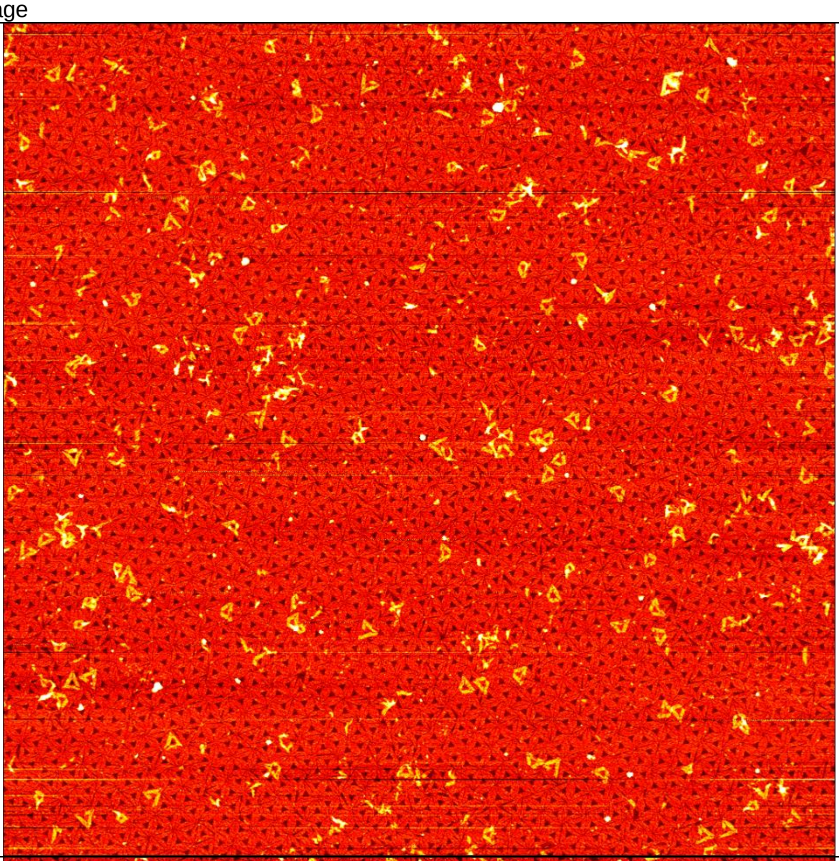	0.37	1.34
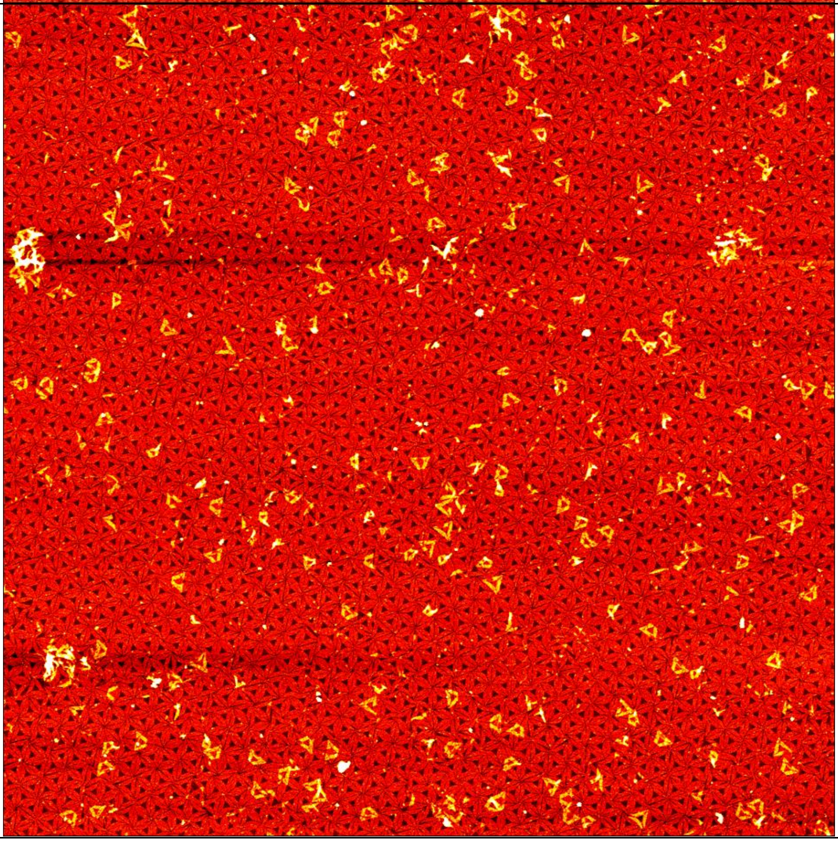	0.40	1.10

Image	p_6	μ_2
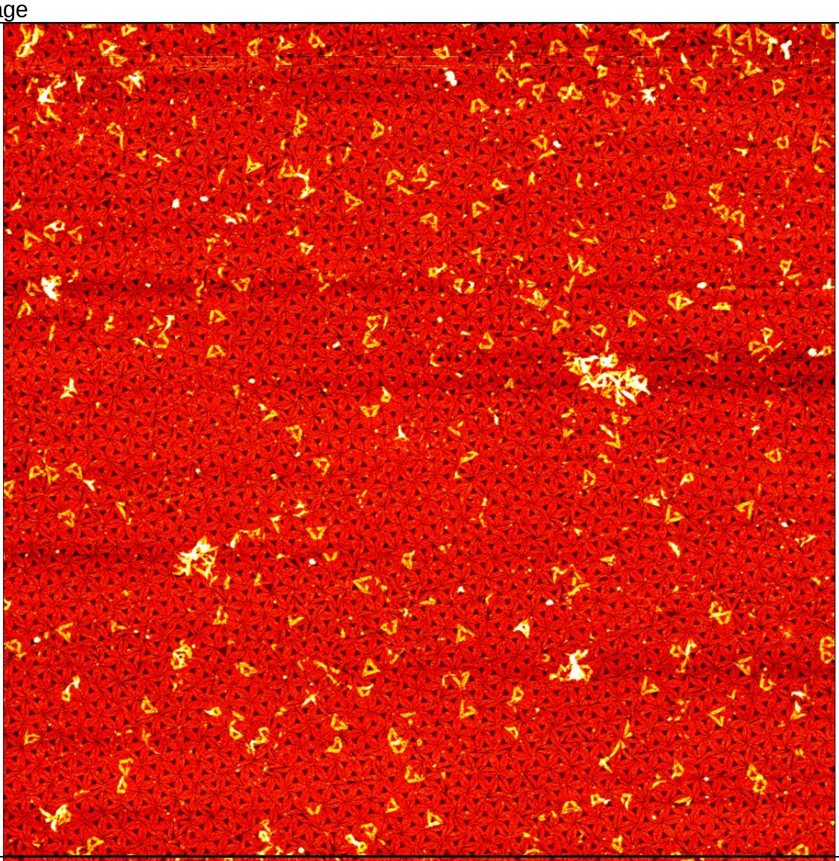	0.36	1.15
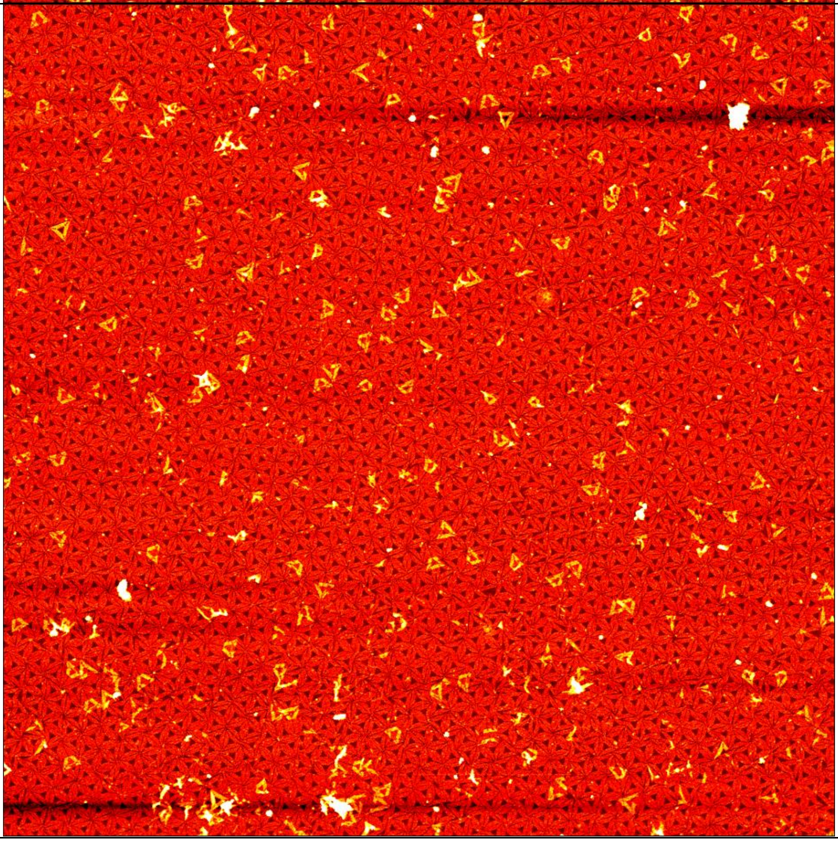	0.37	1.24

Image	p_6	μ_2
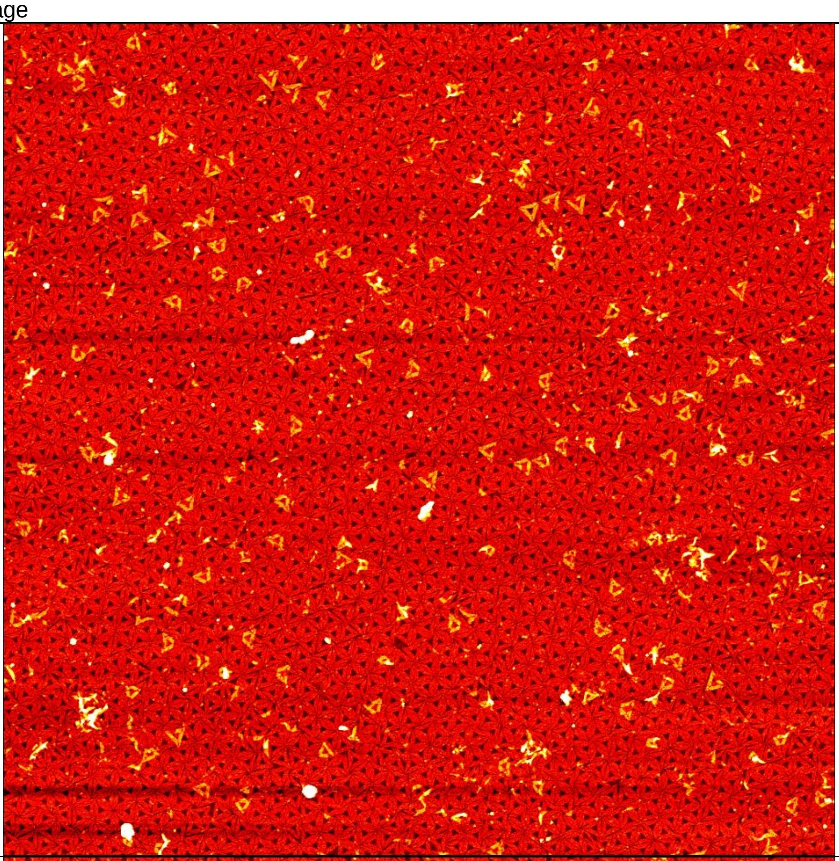	0.40	1.11
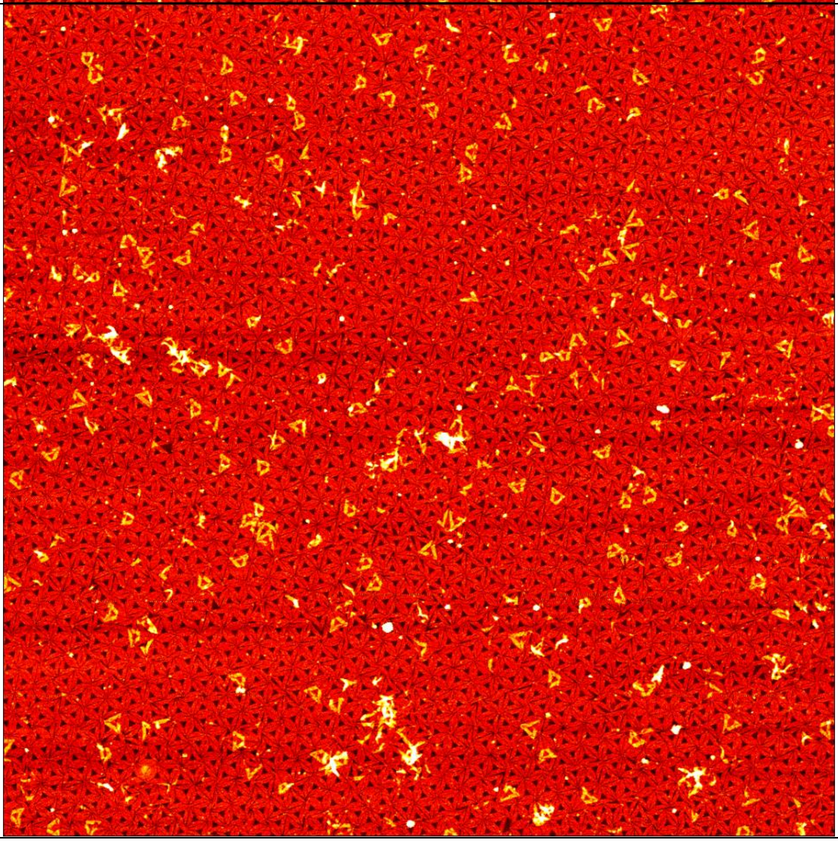	0.39	1.09

Image	p_6	μ_2
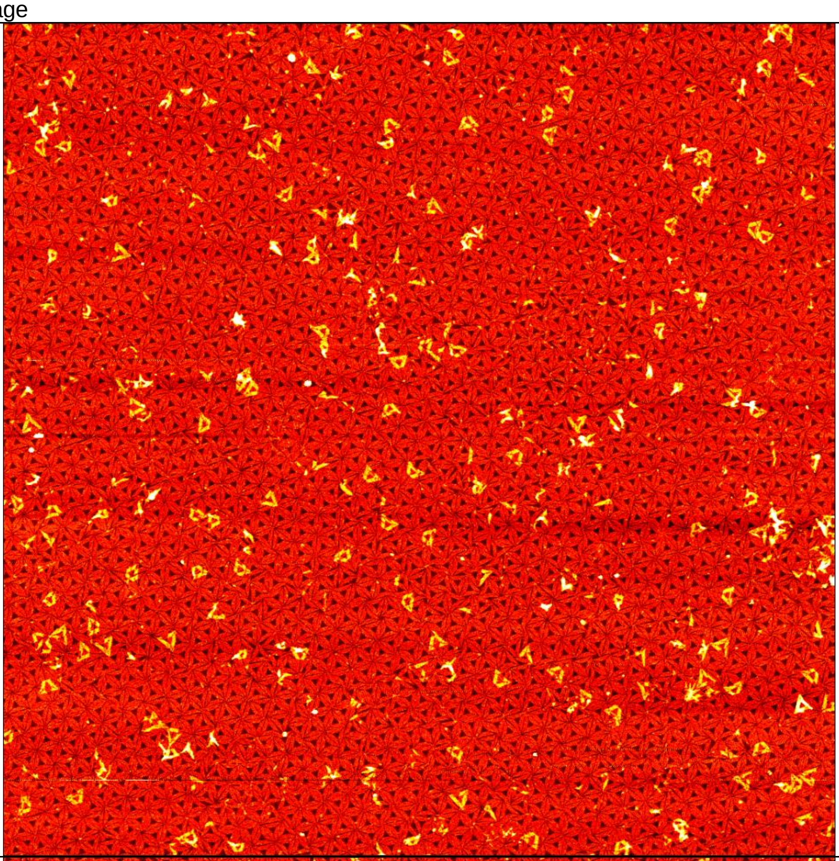	0.40	1.14
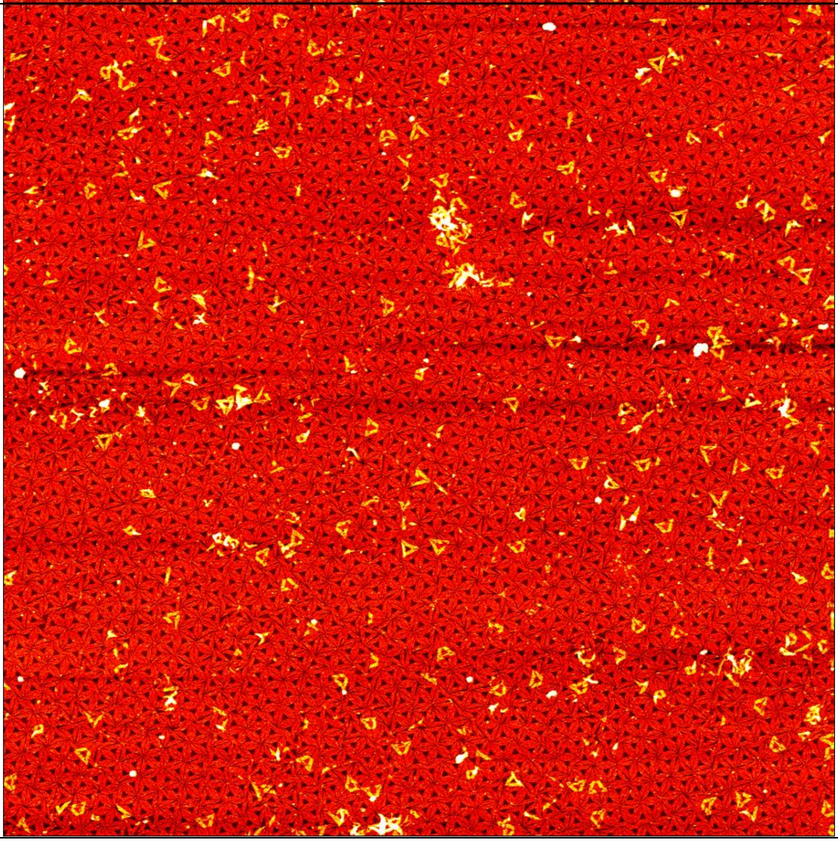	0.39	1.18

Image	p_6	μ_2
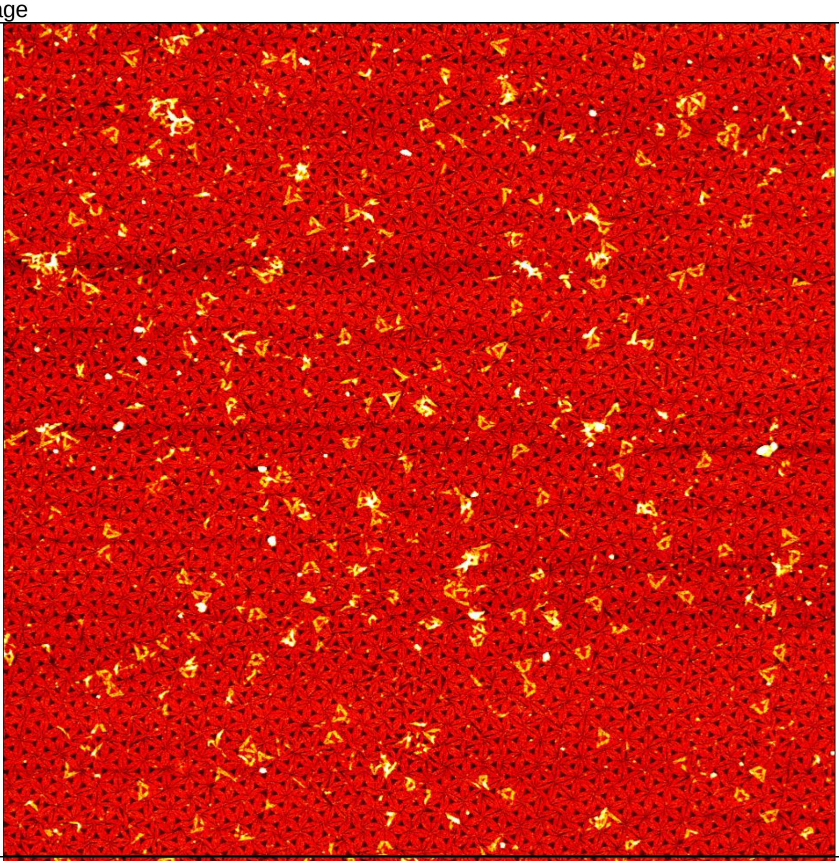	0.40	1.07
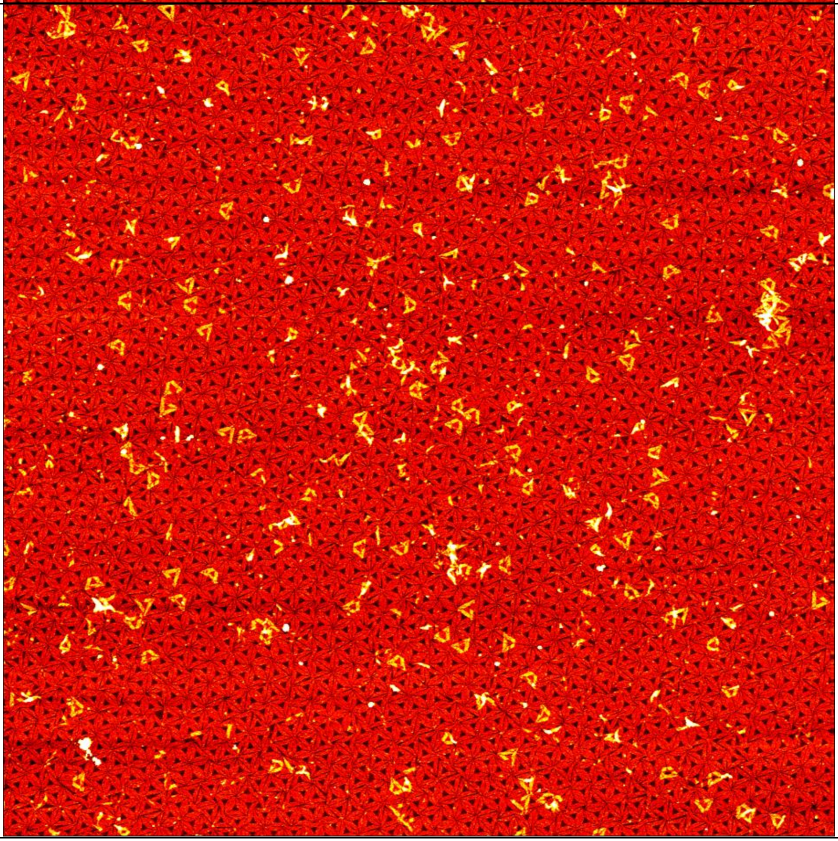	0.38	1.15

Image	p_6	μ_2
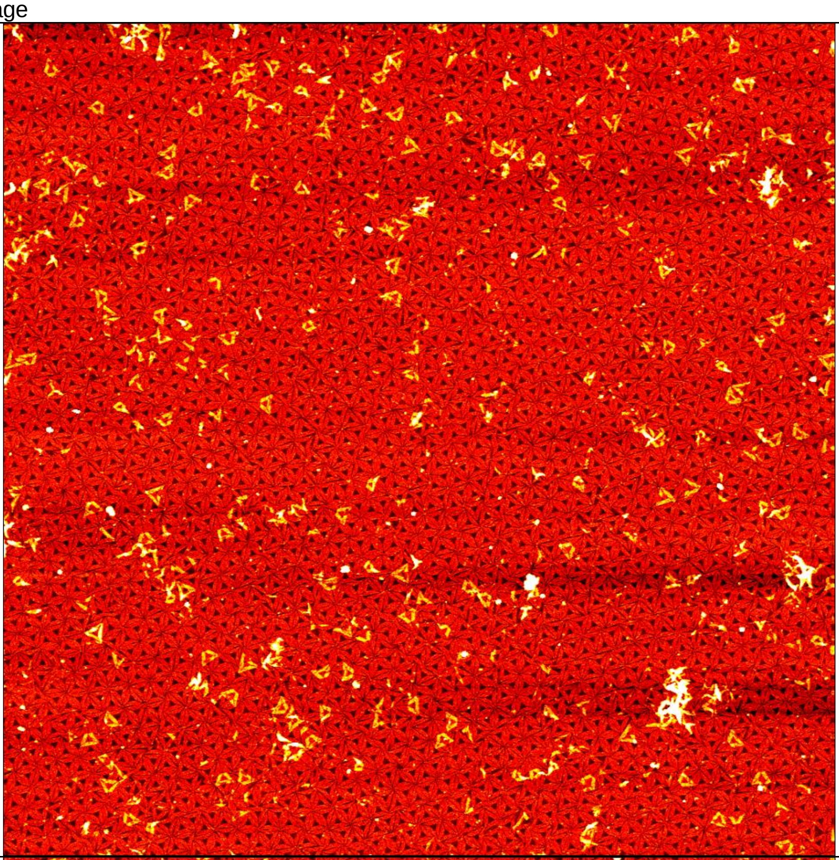	0.37	1.15
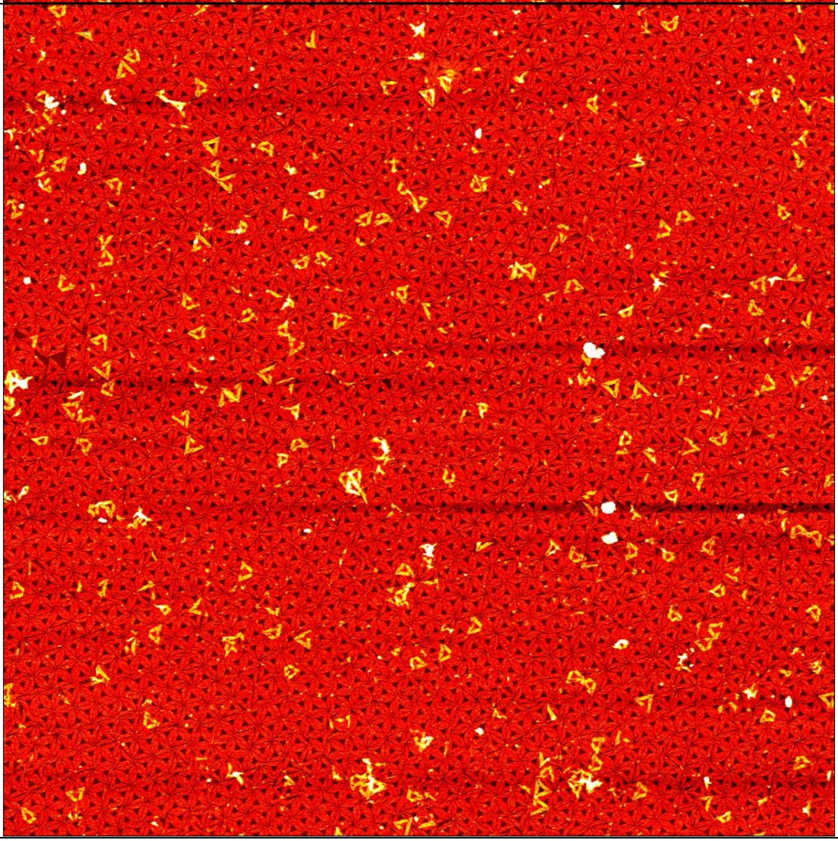	0.40	1.07

Image	p_6	μ_2
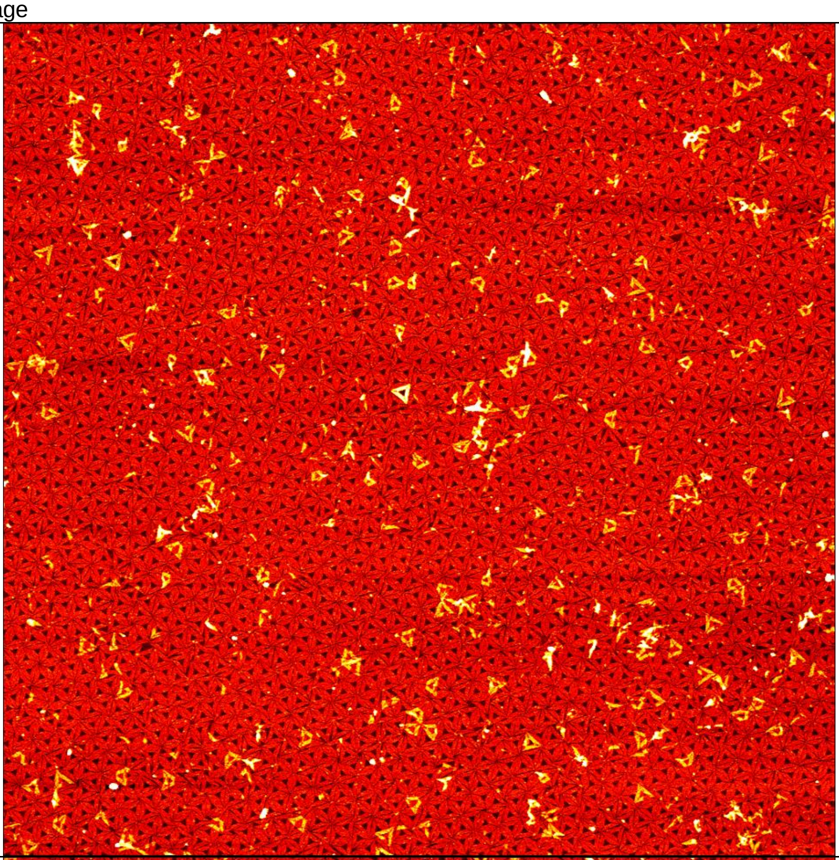	0.40	1.07
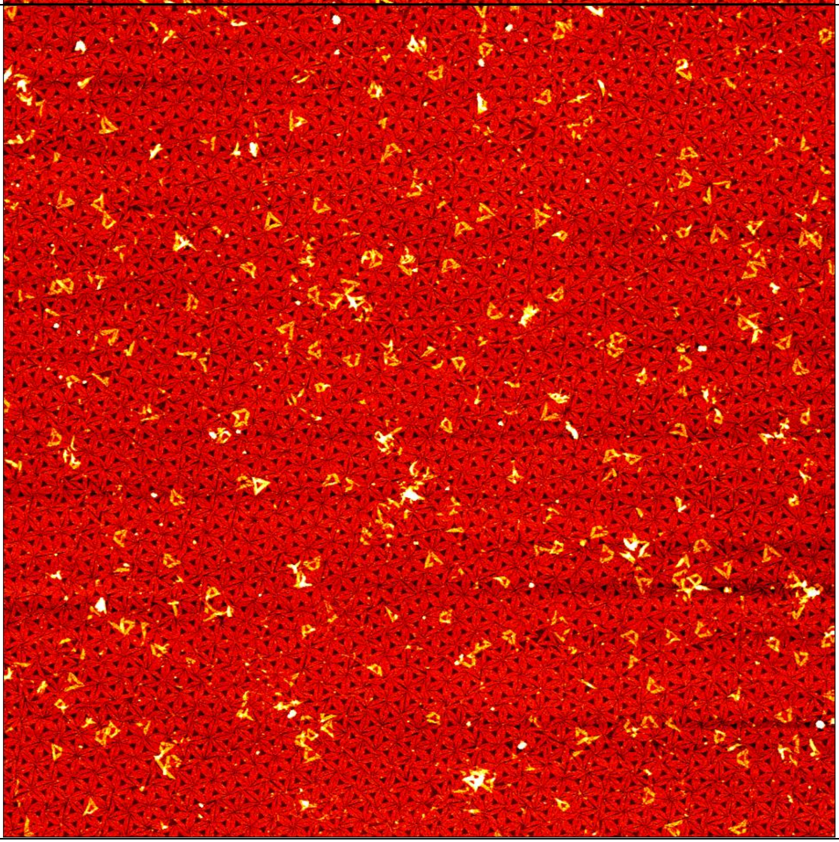	0.38	1.15

Image	p_6	μ_2
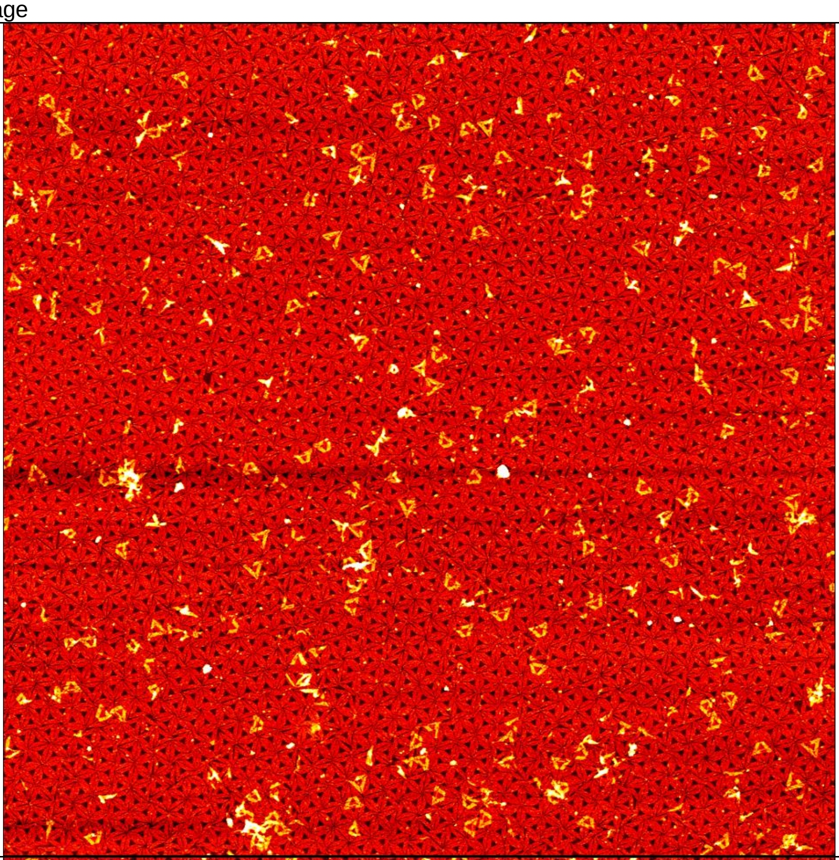	0.40	1.12
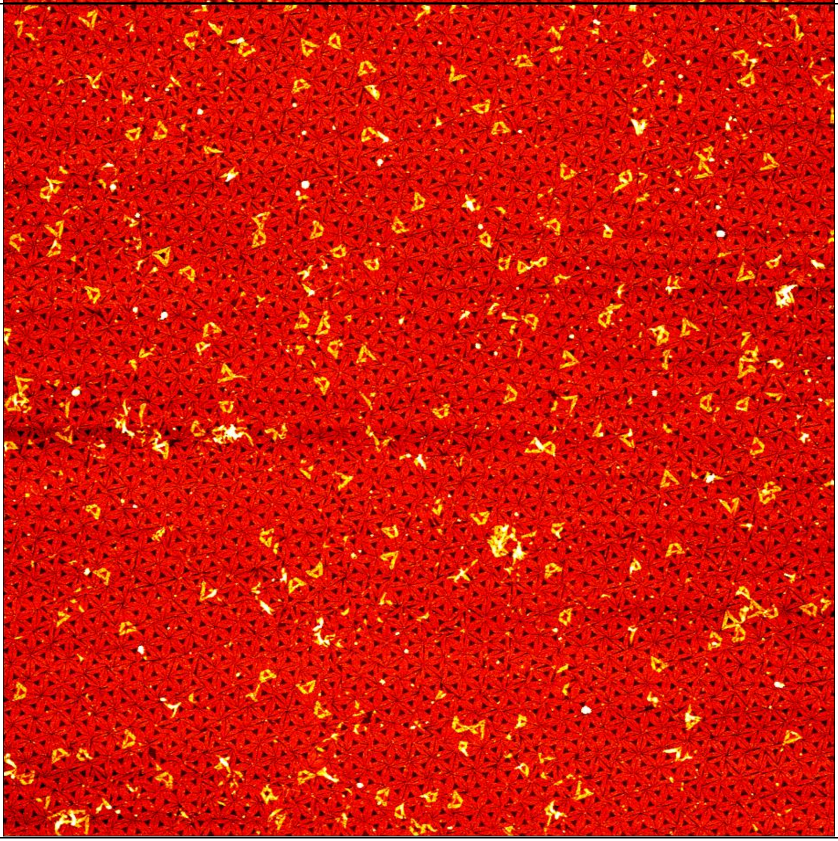	0.41	1.08

Image	p_6	μ_2
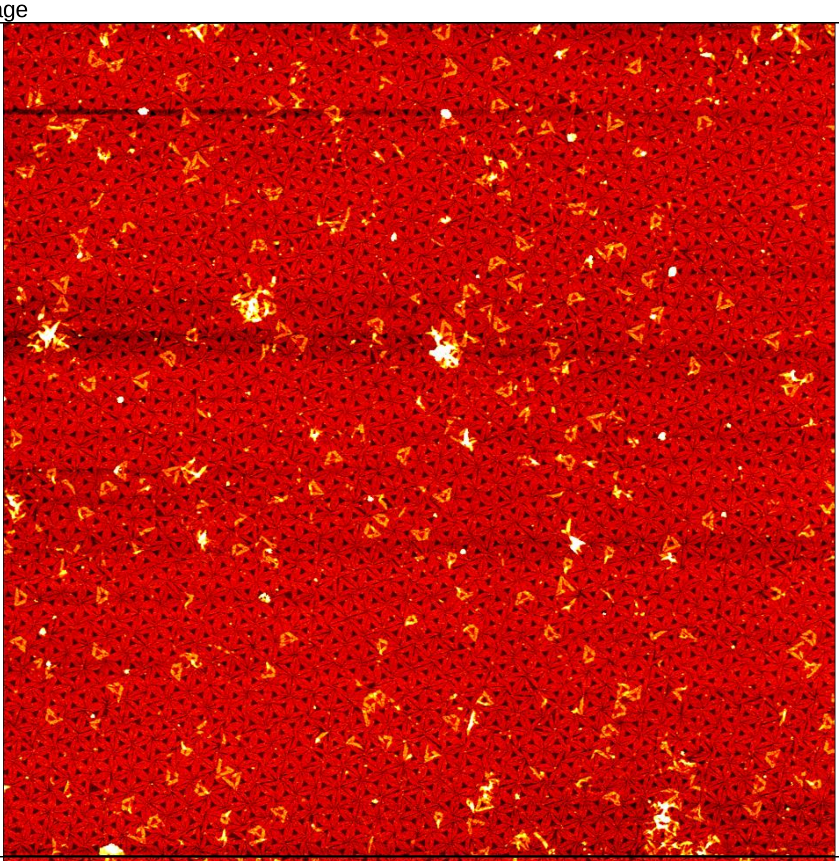	0.39	1.11
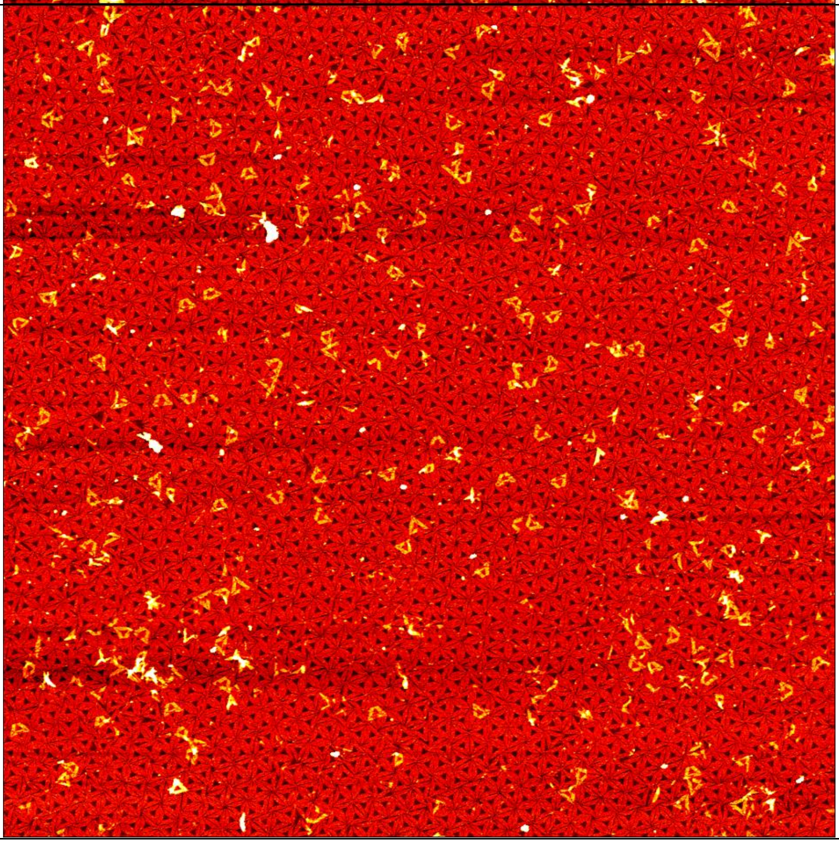	0.39	1.20

Image	p_6	μ_2
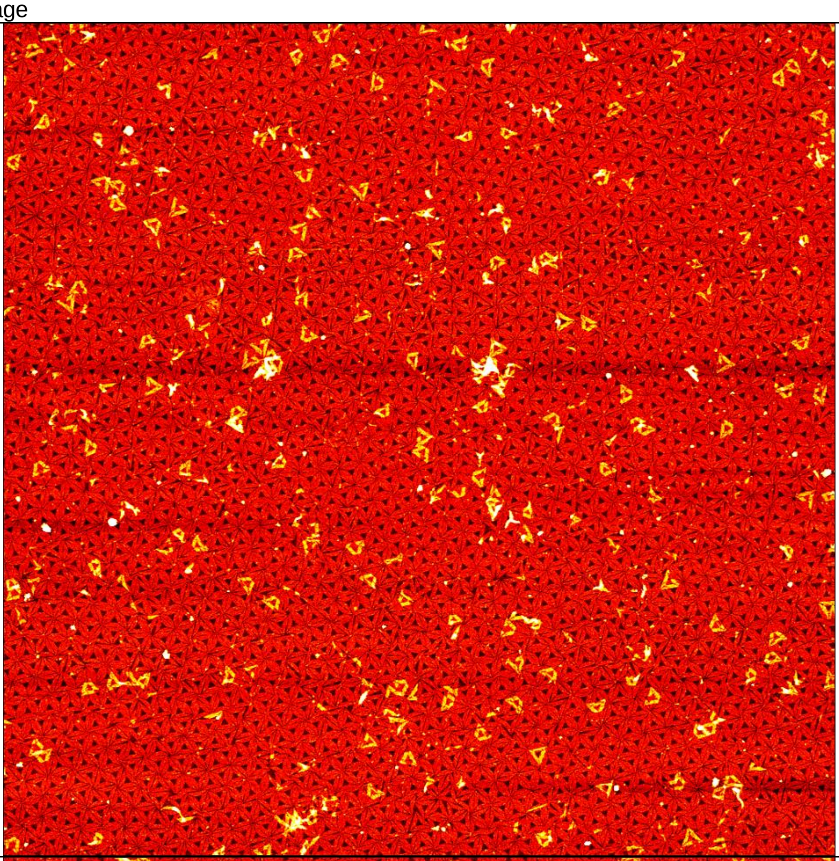	0.39	1.08
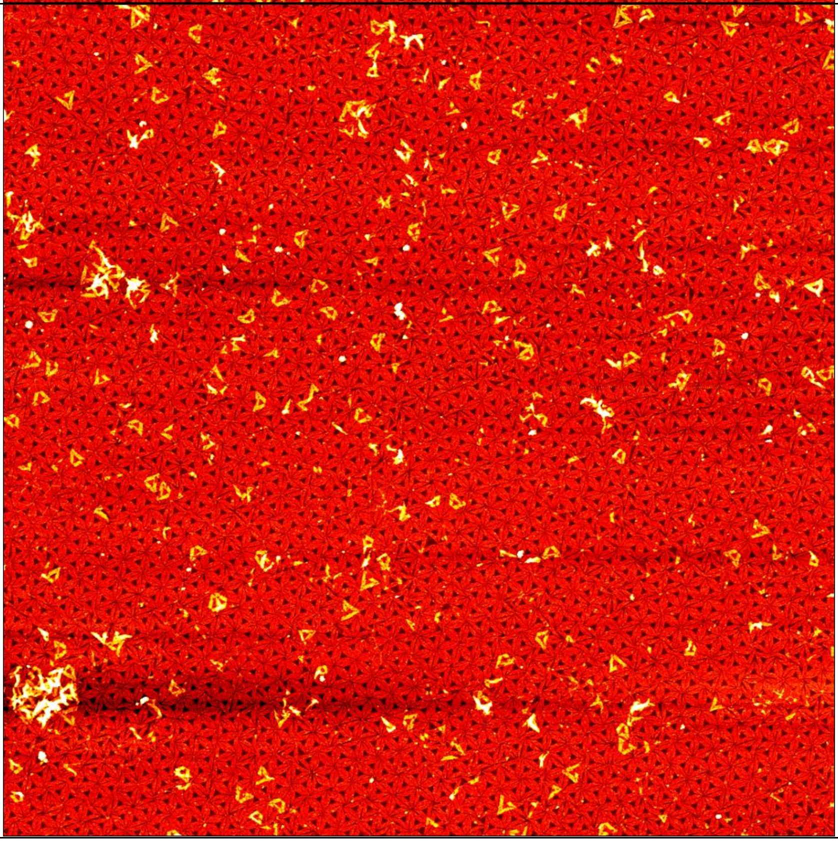	0.41	1.11

Image	p_6	μ_2
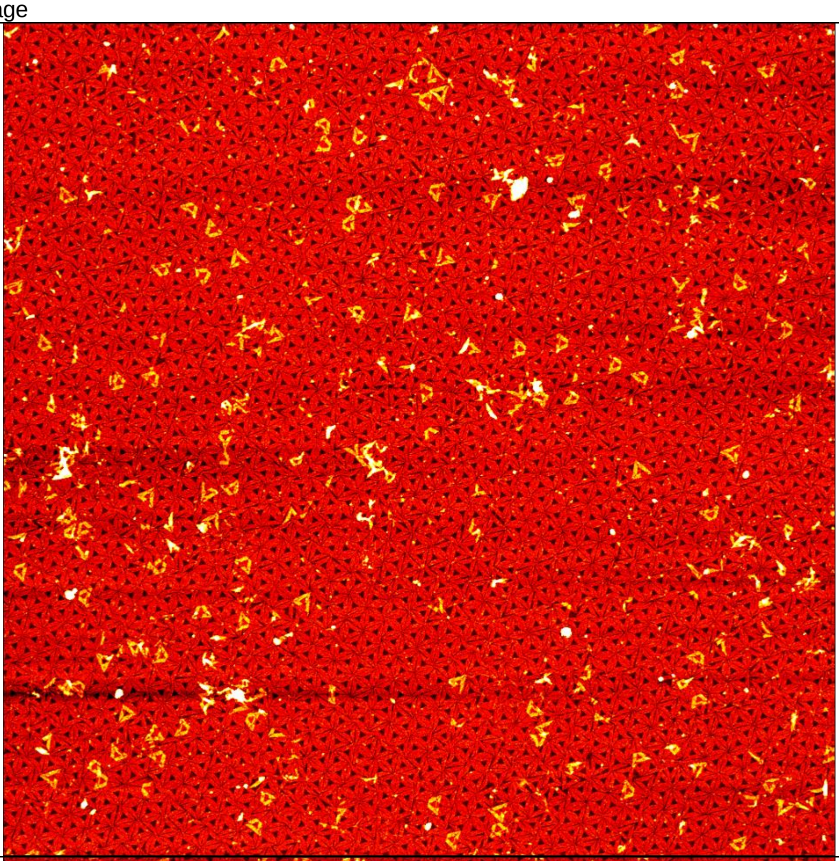	0.37	1.12
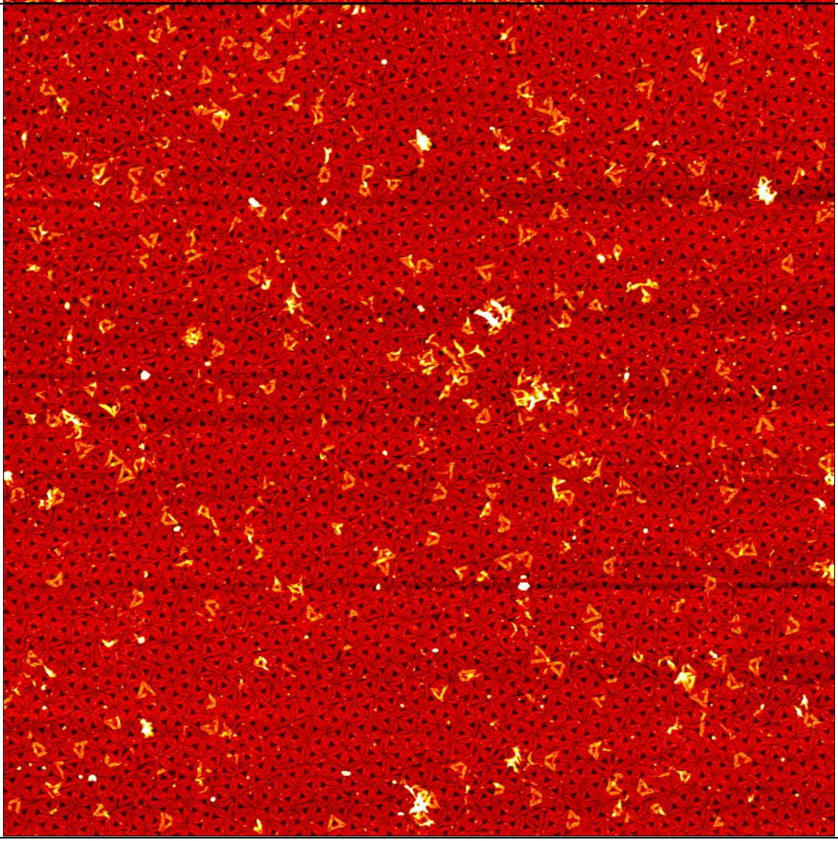	0.39	1.13

Image	p_6	μ_2
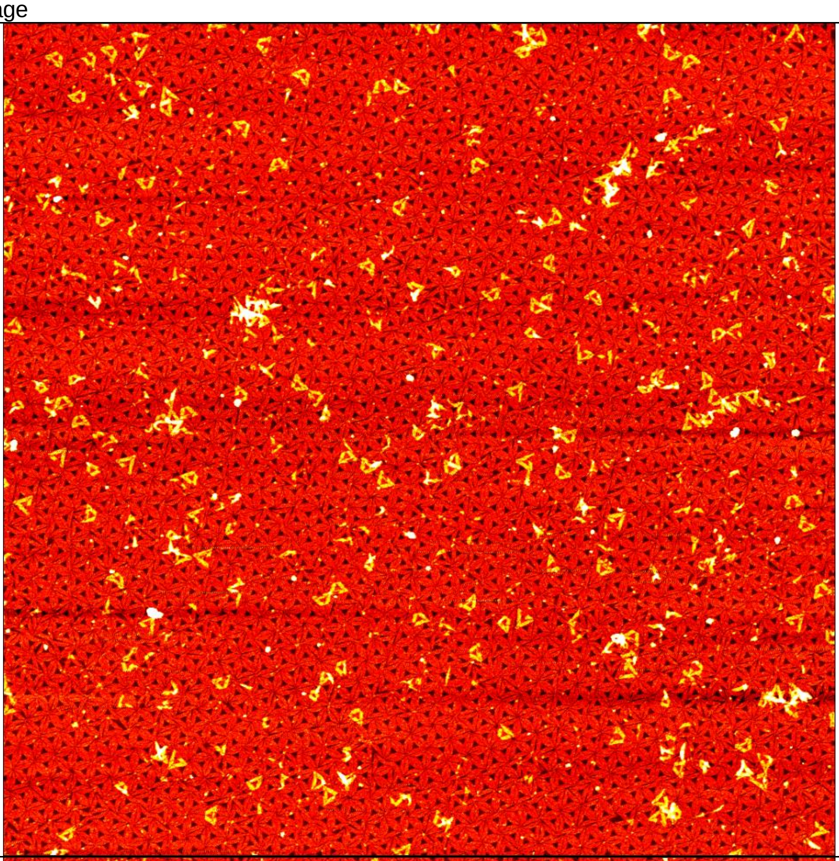	0.40	1.10
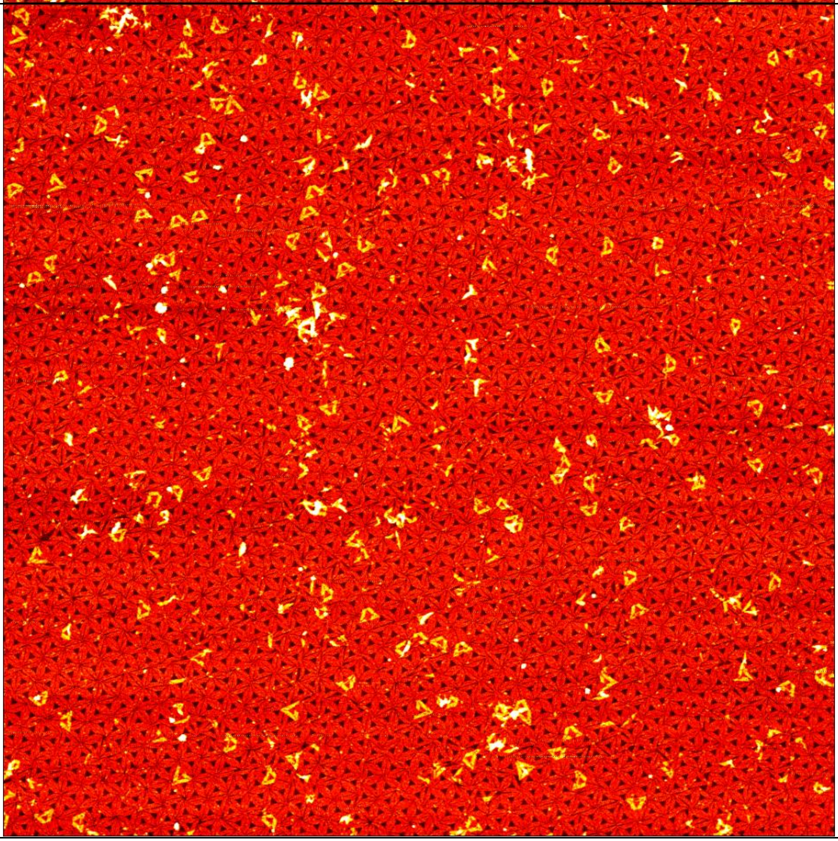	0.39	1.12

Image	p_6	μ_2
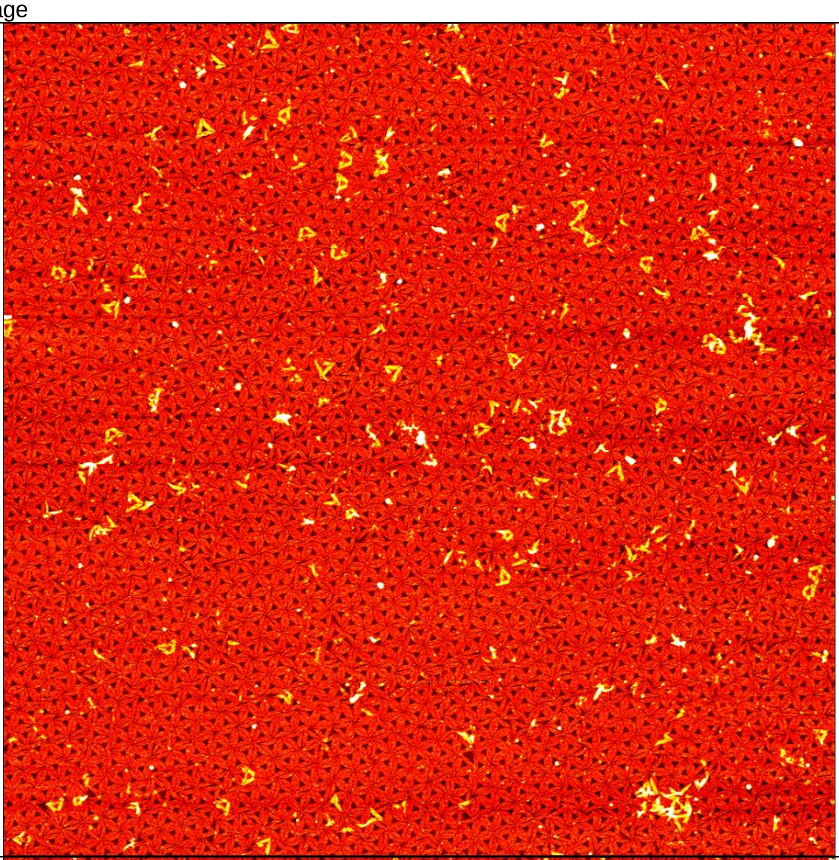	0.40	1.04
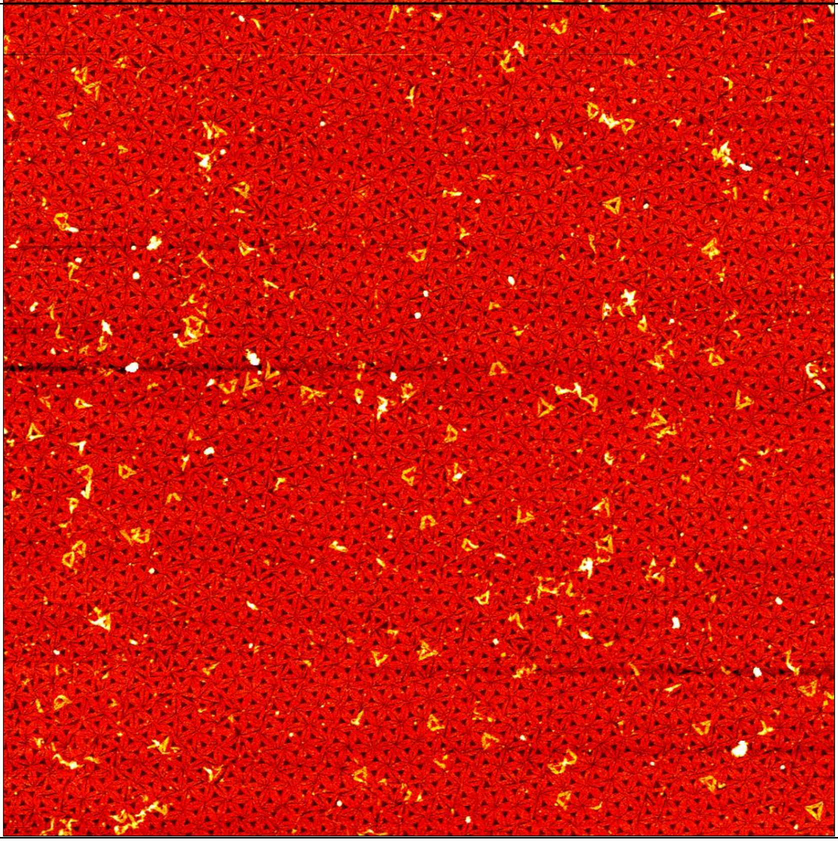	0.39	1.09

Image	p_6	μ_2
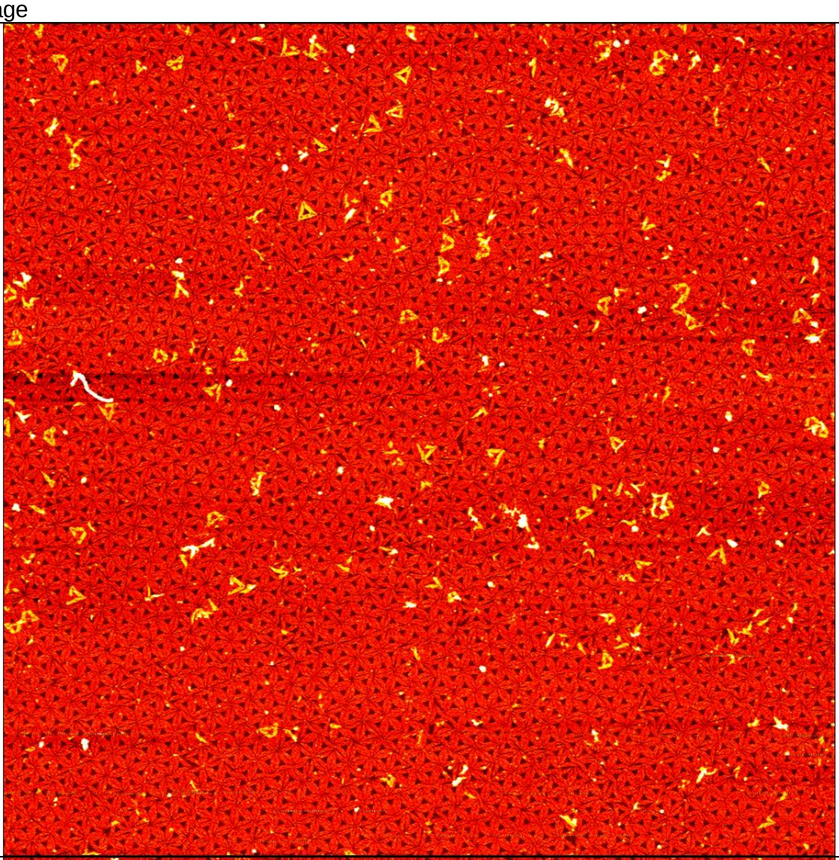	0.40	1.05
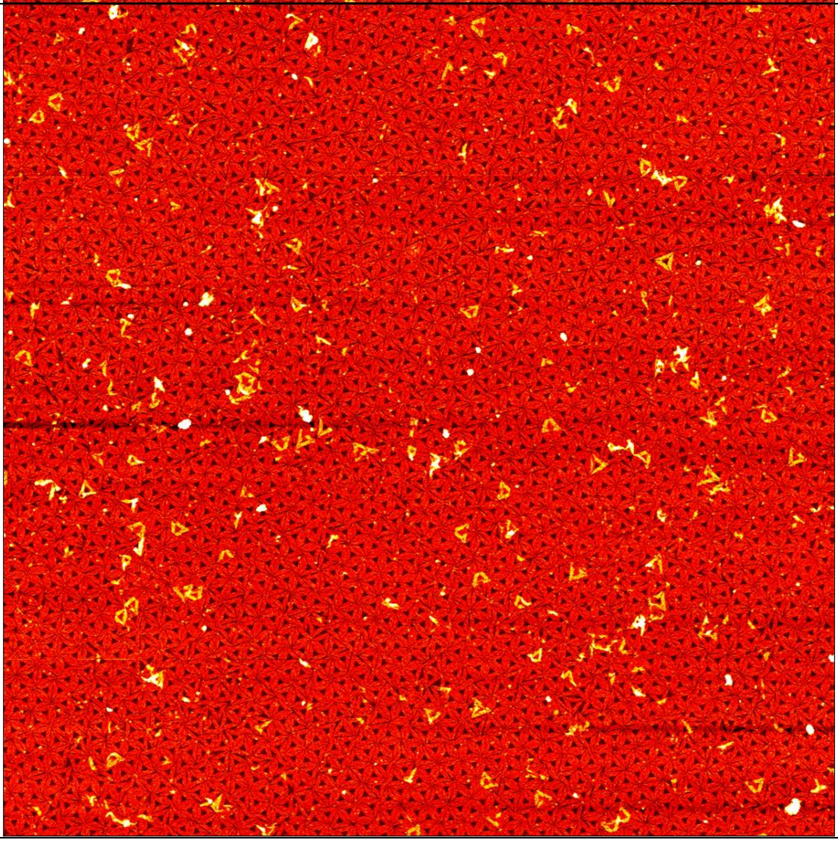	0.40	1.08

Image	p_6	μ_2
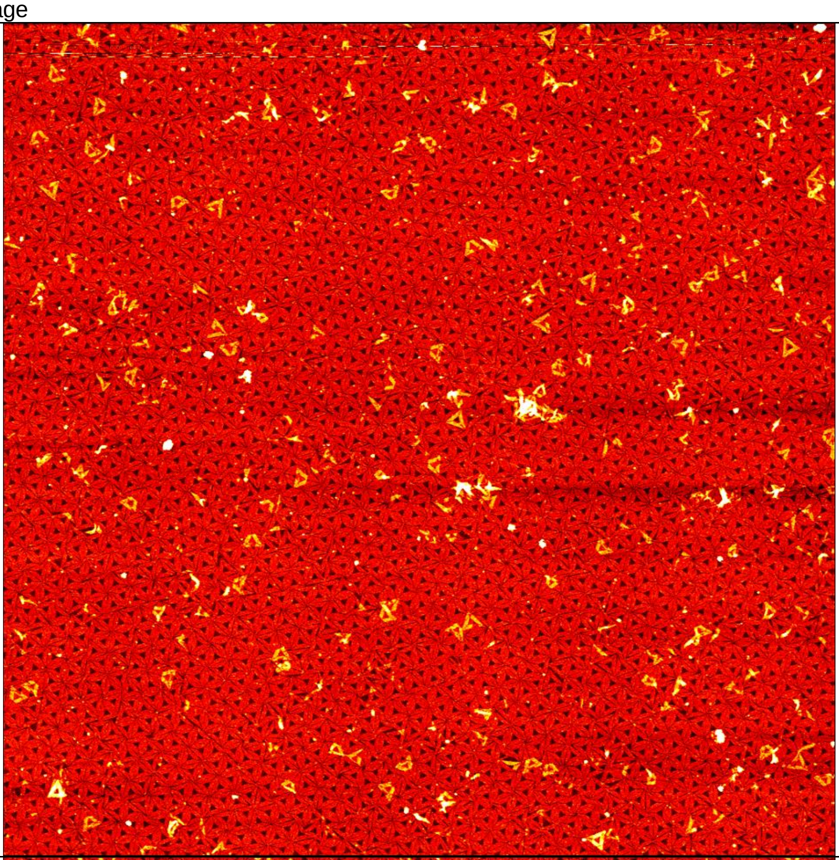	0.39	1.10
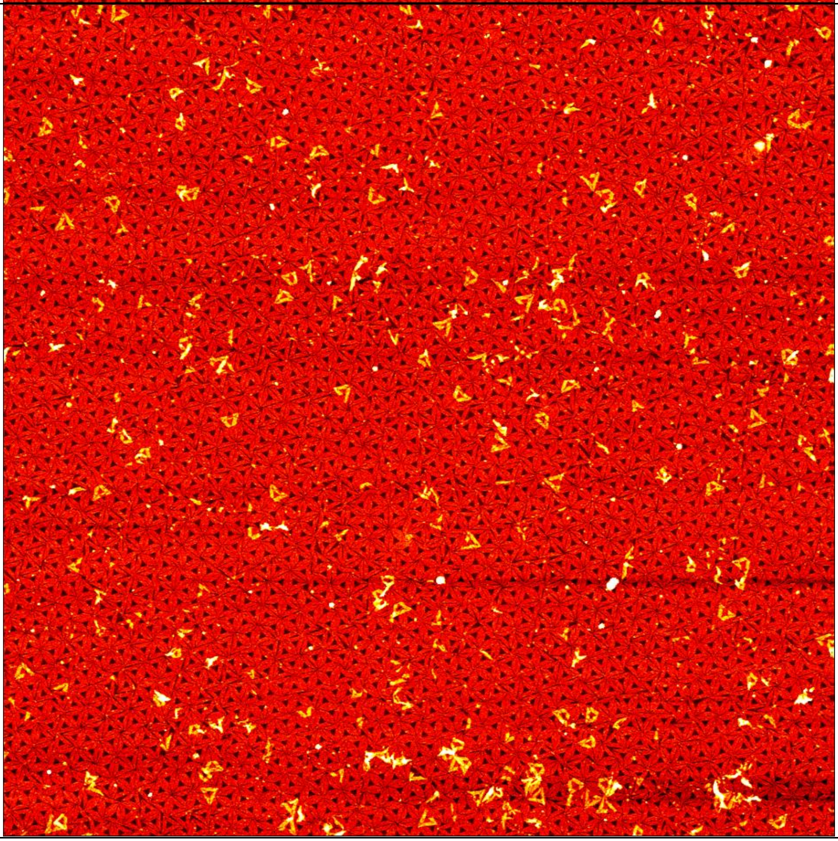	0.39	1.15

Image	p_6	μ_2
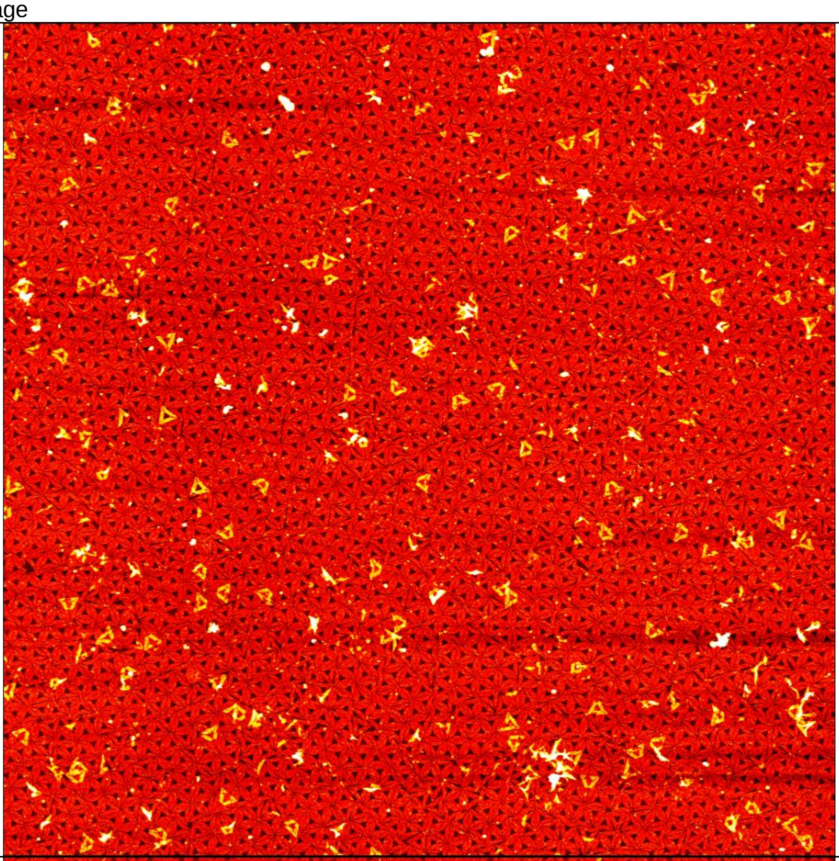	0.40	1.16
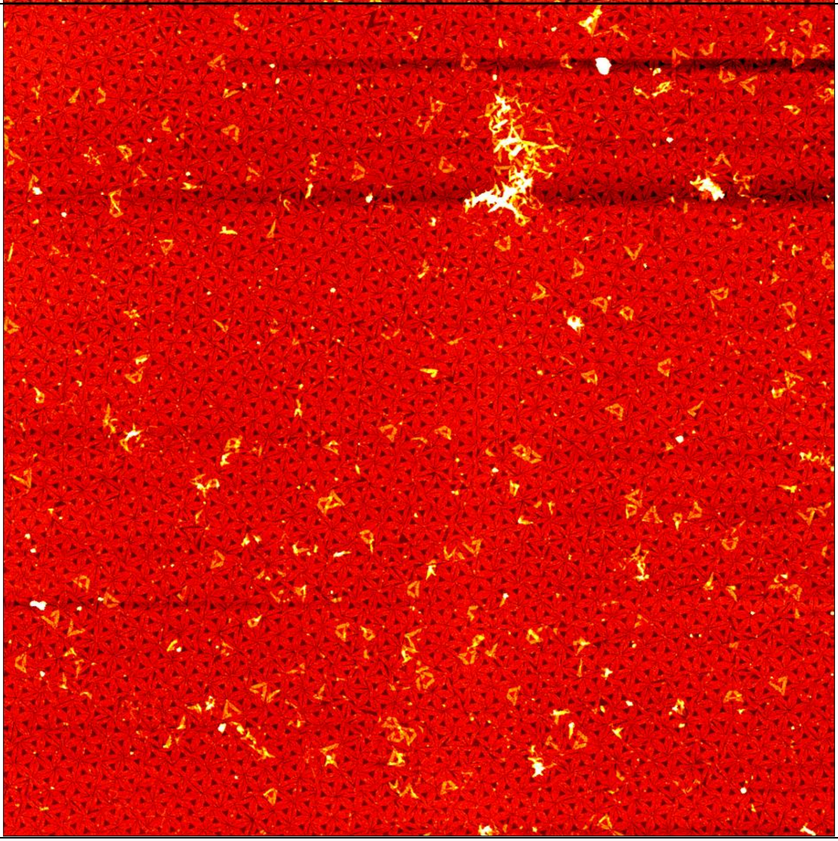	0.39	1.09

Image	p_6	μ_2
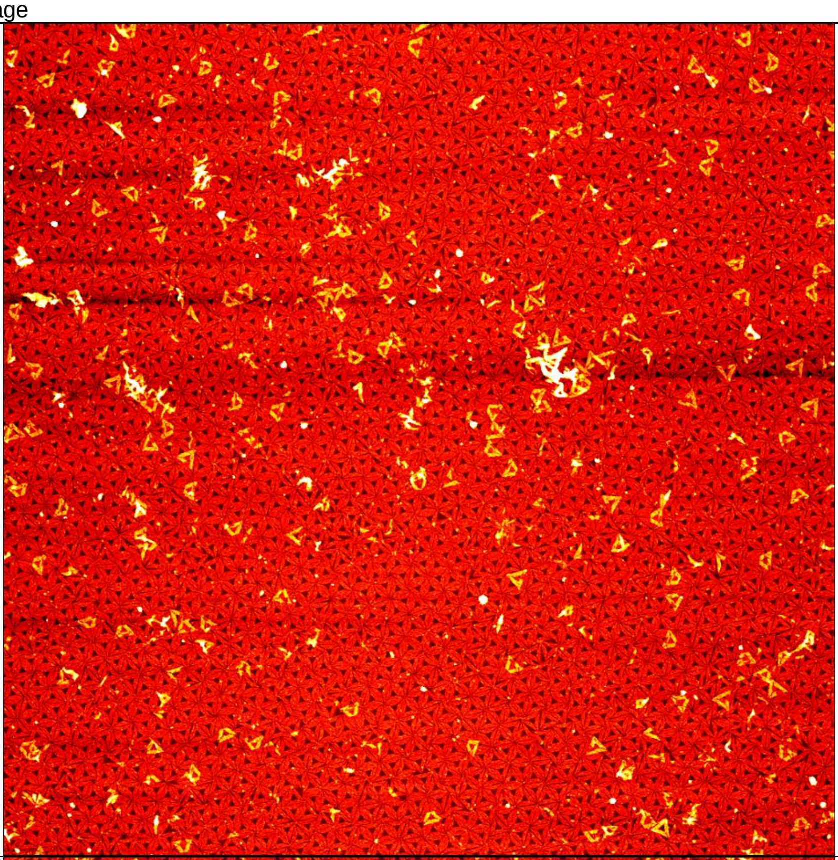	0.38	1.20
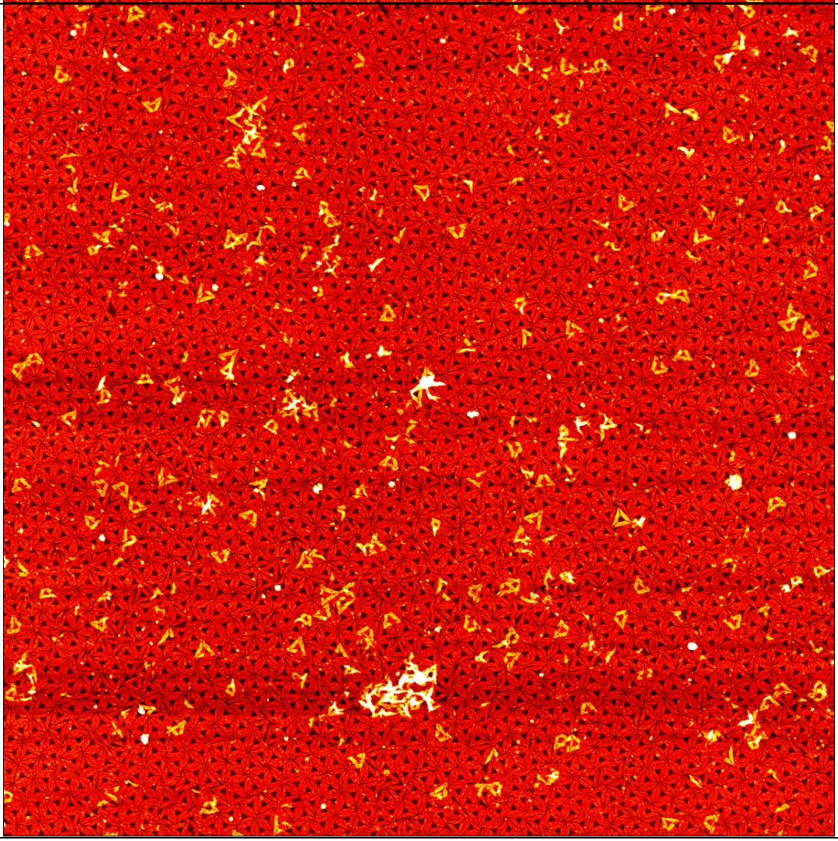	0.39	1.12

Image	p_6	μ_2
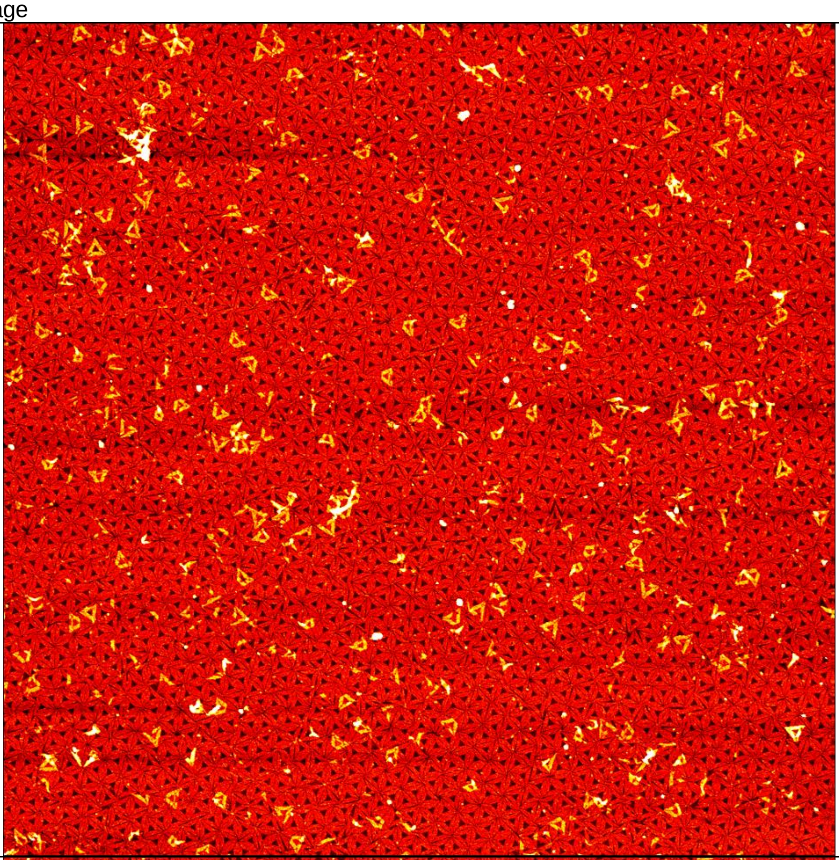	0.38	1.13
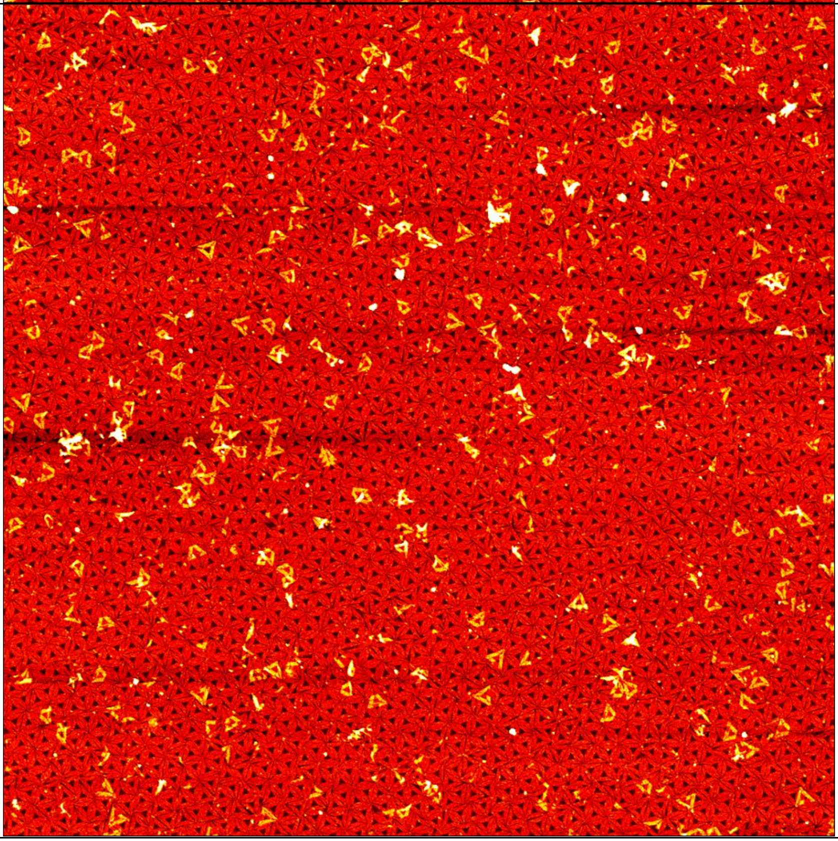	0.38	1.19

Image	p_6	μ_2
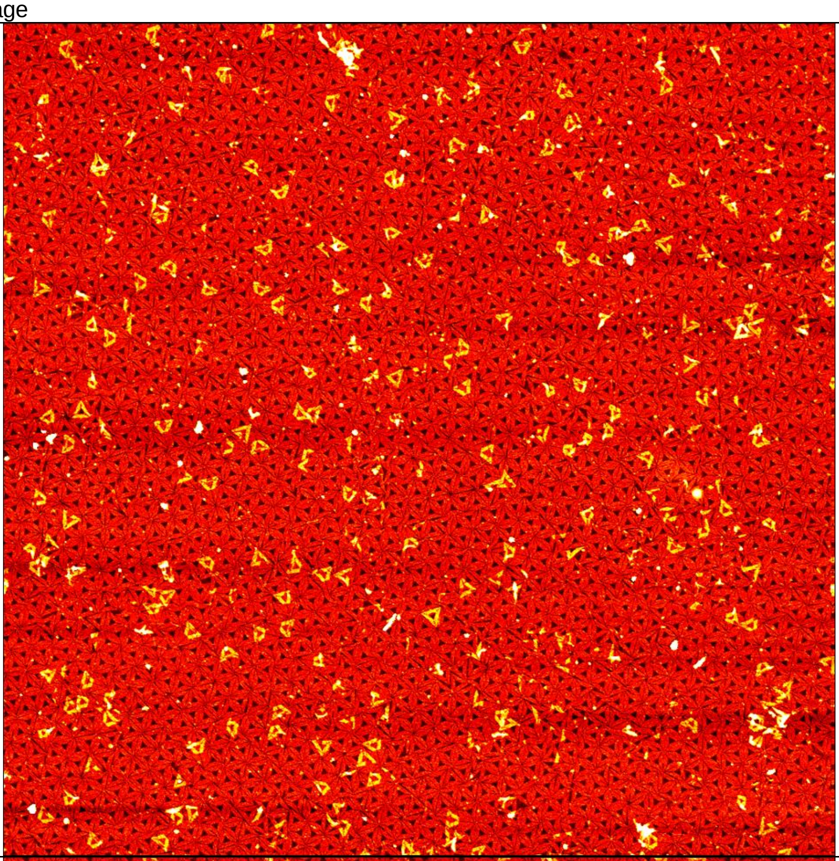	0.40	1.06
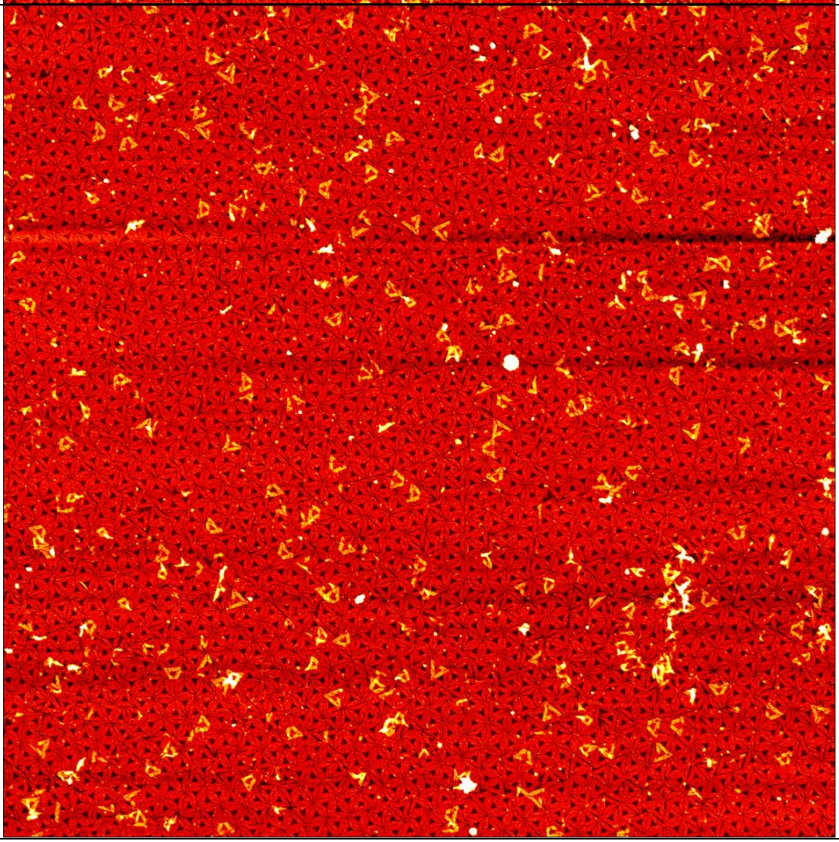	0.38	1.14

Image	p_6	μ_2
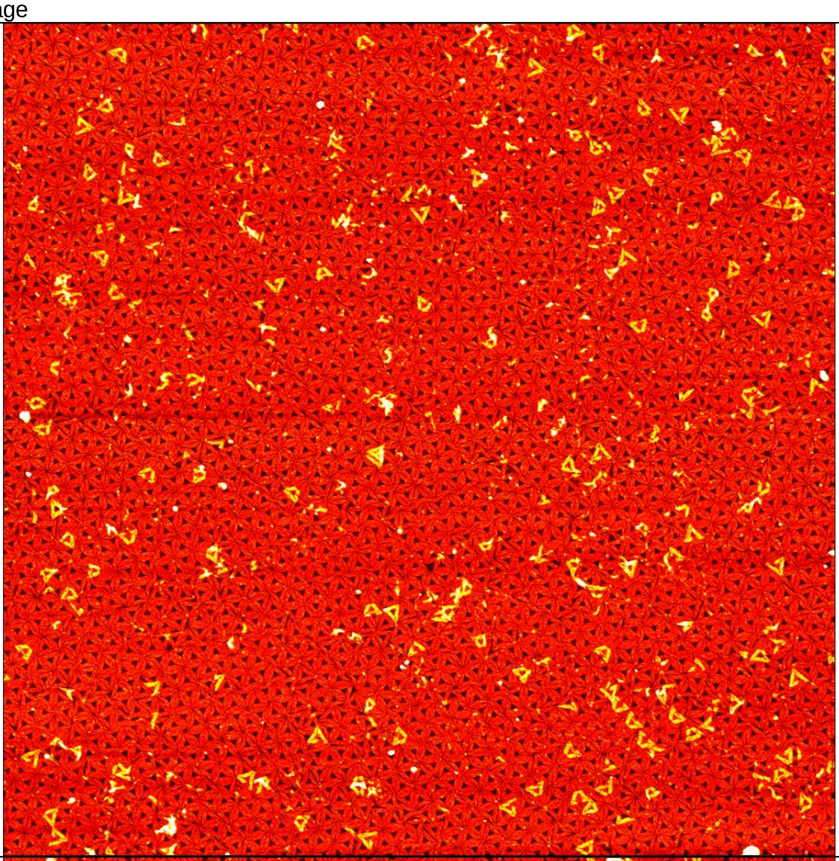	0.39	1.12
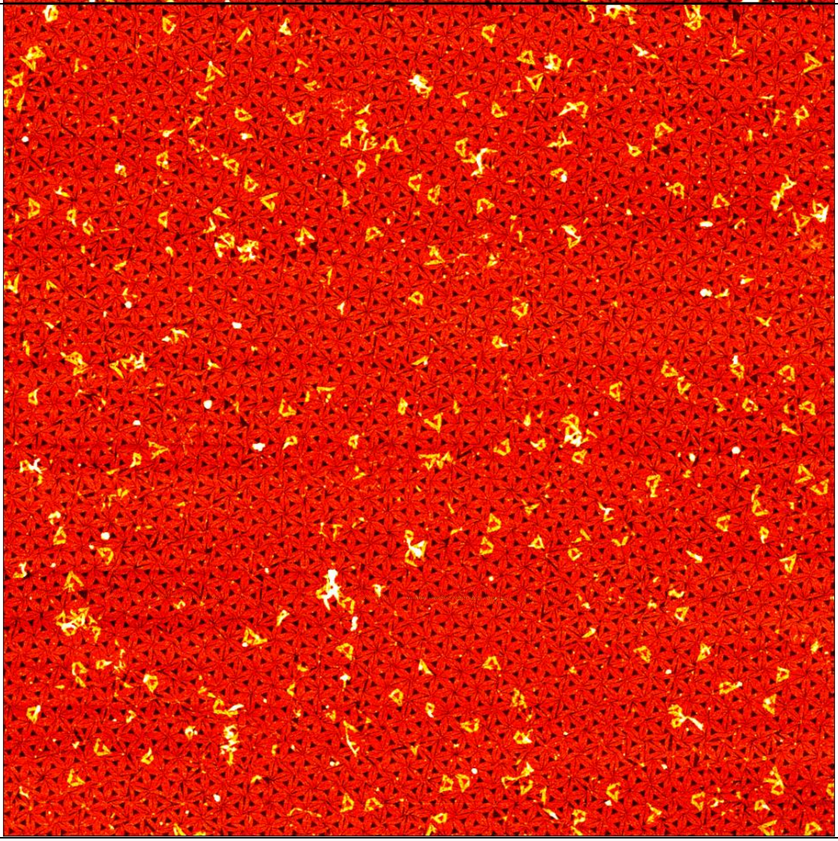	0.40	1.08

Image	p_6	μ_2
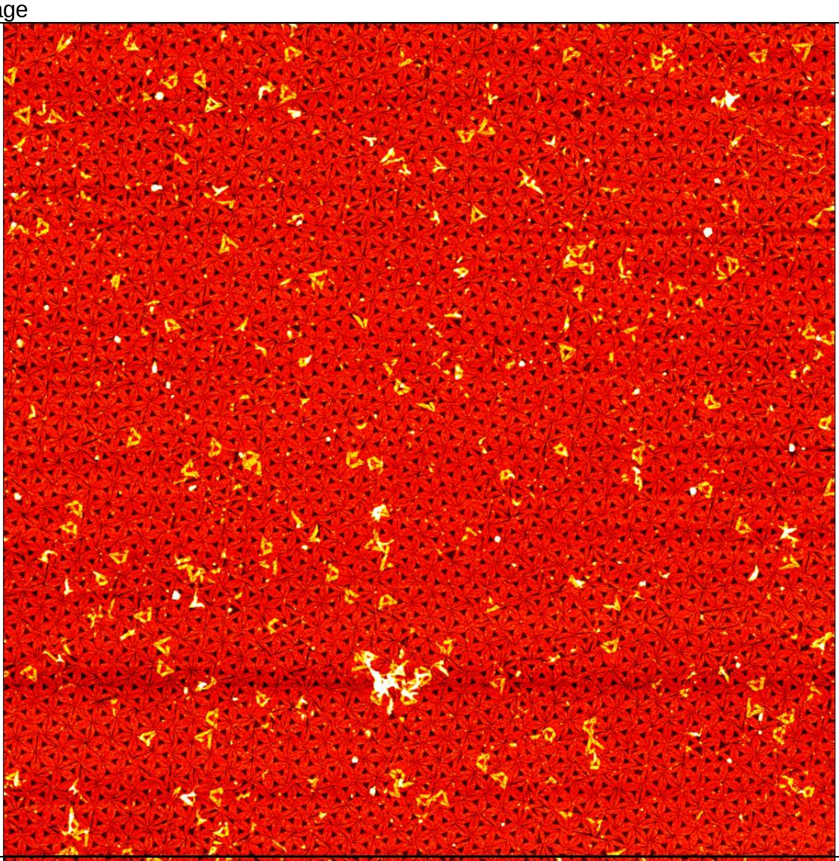	0.39	1.09
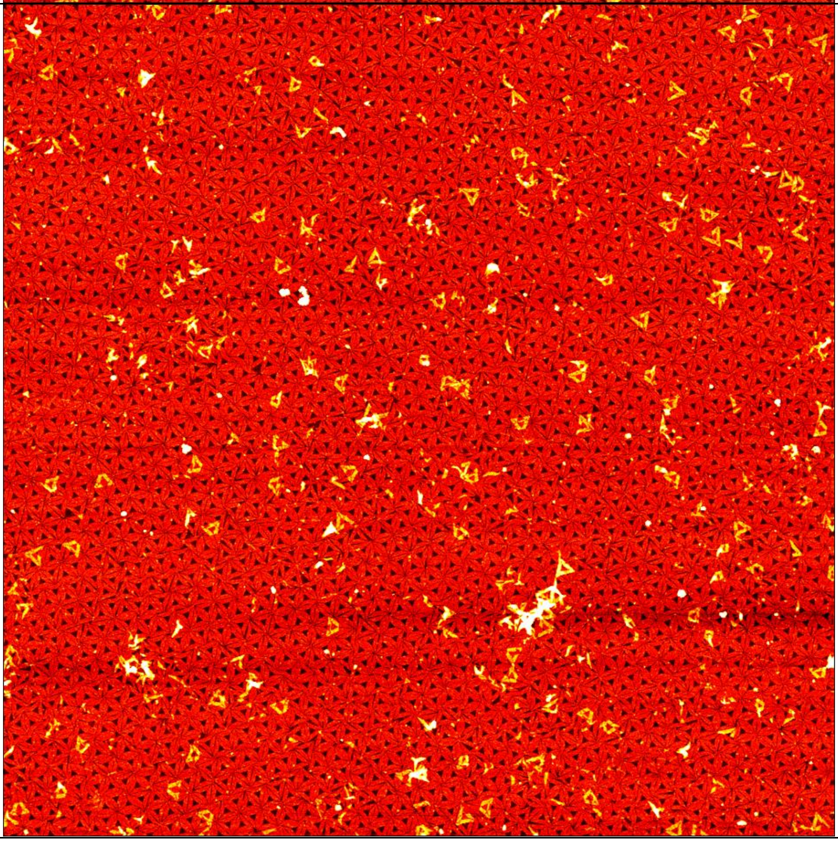	0.38	1.12

Image	p_6	μ_2
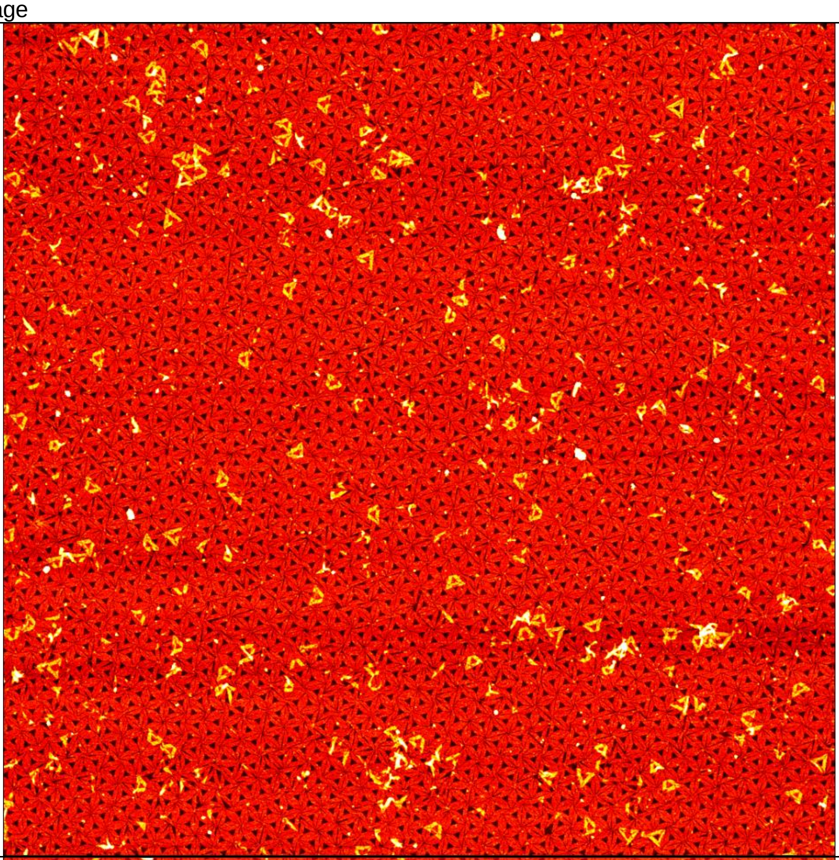	0.39	1.08
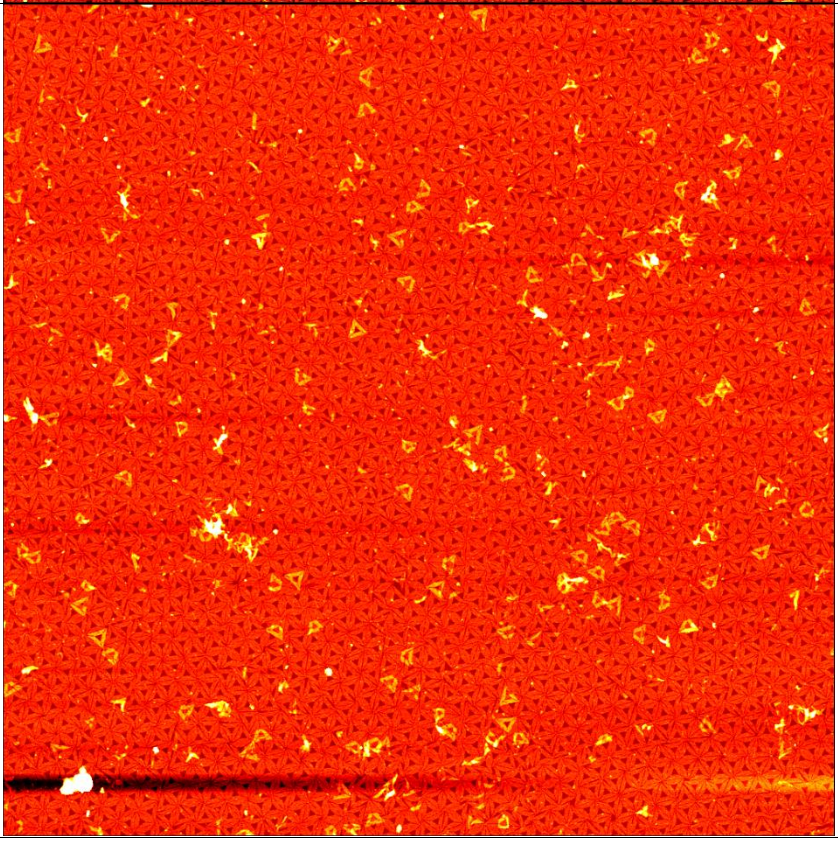	0.39	1.11

Image	p_6	μ_2
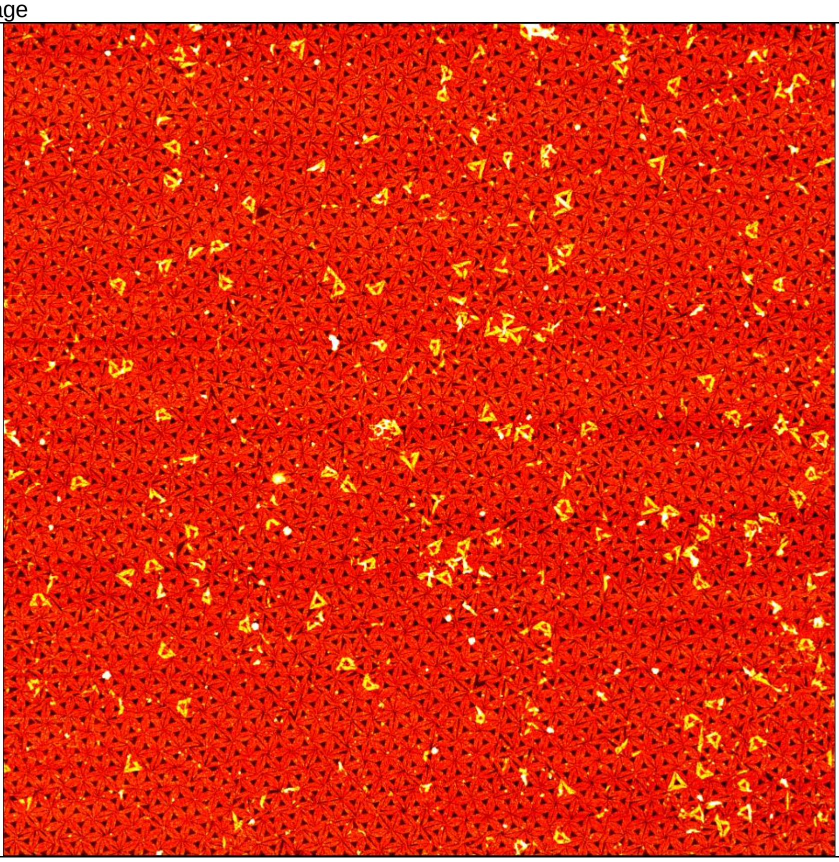	0.39	1.11

Author Contributions

Yang Xin
Conceptualization: Supporting, Data curation: Equal, Formal analysis: Equal, Investigation: Equal, Methodology: Equal, Validation: Equal, Visualization: Equal, Writing – original draft: Supporting, Writing – review & editing: Equal

Boxuan Shen
Conceptualization: Supporting, Data curation: Equal, Formal analysis: Equal, Investigation: Equal, Validation: Equal, Visualization: Equal, Writing – original draft: Supporting, Writing – review & editing: Equal

Mauri A. Kostiainen
Supervision: Supporting, Writing – review & editing: Equal

Guido Grundmeier
Supervision: Supporting, Writing – review & editing: Equal

Mario Castro
Data curation: Equal, Formal analysis: Lead, Methodology: Equal, Validation: Equal, Visualization: Equal, Writing – review & editing: Equal

Veikko Linko
Conceptualization: Lead, Methodology: Equal, Supervision: Supporting, Writing – original draft: Supporting, Writing – review & editing: Equal

Adrian Keller
Conceptualization: Lead, Data curation: Equal, Formal analysis: Equal, Methodology: Equal, Validation: Equal, Visualization: Equal, Supervision: Lead, Writing – original draft: Lead, Writing – review & editing: Equal
